# Analysis of spatial characteristics and influencing factors of the flow network of highly educated talents from national and local perspective

**DOI:** 10.1038/s41598-024-60436-5

**Published:** 2024-04-26

**Authors:** Wentian Shi, Wenlong Yang, Xueying Mu, Fan Yang

**Affiliations:** 1 Department of Hospitality Management, Shanghai Business School, Shanghai, 200235 China; 2 Institute of World Economy, Shanghai Academy of Social Sciences, Shanghai, 200020 China; 3 School of Philosophy and Social Development, South China Normal University, Guangzhou, 510631 China; 4 Institute of Information, Shanghai Academy of Social Sciences, Shanghai, 200235 China

**Keywords:** Highly educated talents, Spatial flow, Complex network, Influencing factors, Yangtze River Delta region, Socioeconomic scenarios, Sustainability, Environmental social sciences, Environmental economics

## Abstract

Based on dynamic monitoring data on China’s population, by using complex networks, spatial analysis and mathematical measurement, this study reveals the spatial characteristics and influencing factors of the network of flows of highly educated talents in the Yangtze River Delta region from the national and local perspectives. In the two perspectives, the network has strong isomorphism and certain differences. The in-flow of highly educated talents from cities with high administrative levels and more developed economies to Shanghai constitutes the core of the entire network. From a national perspective, highly educated talents tend to converge to the Yangtze River Delta region. From a local perspective, it was found that these talents cluster towards a limited number of cities in the region. From both perspectives, the flow network has developed into a “core-periphery” progressive hierarchical structure, with Shanghai becoming the sole core city. There is little difference in the influencing factors of talent mobility from both macro and meso perspectives. Highly educated talents would frequently flow between cities with strong economic development levels, and cities with high education level, scientific and technological level, complete infrastructure, and good aesthetics. However, geographical distance still plays a hindering role in the flow of highly educated talents, and factors such as cultural identity, institutional, and social modality differences among regions also have a certain effect on the flow of these talents.

## Introduction

The dynamic of big cities is constantly calling for talents, who can bring their expertise into the market and make them more prosperous. Talents are the top resource driving urban development especially highly educated ones. These talents are the most active and dynamic production factor for the wealth of cities, so their flows have become an important symbol of urban vitality^1^. The flow of highly educated talents can effectively facilitate regional knowledge diffusion, technological progress, and economic growth, and also affects the formation and transfer of global or regional knowledge, technology, and economic hubs^2–6^. The competitiveness of a city depends largely on the market operation for highly educated talents, so cultivating and attracting more people from this group is a key factor affecting whether a city can succeed in international competitions^7^. With the intensified global economic integration, the scale and frequency of flows of highly educated talents have risen sharply around the world, and almost all countries and regions have realized the importance of attracting these talents to leverage their positions in international competitions^1,8^. They are all actively trying to create highlands for highly educated talents, so an unprecedented war for winning the attraction of these talents has quietly started and the competition has become increasingly fierce^9,10^. In the reshuffling process of urban structures in China, the “war for talents” has entered a white-hot stage, with many cities turning their attention to highly educated talents. In order to firmly attract such talents, all cities, including old first-tier ones, newly emerging ones, and other second- or third-tier regions, have announced various preferential policies for highly educated talents in such fields as housing, household registration, and entrepreneurial support. For example, Beijing, Shanghai, Guangzhou, and Shenzhen have provided many favorable policies for international students and graduate-level talents^11,12^.

High mobility is one of the most significant characteristics of highly educated talents^4^ and this feature is particularly prominent in those still navigating the early stages of their careers^13,14^. The mobility flow of these talents have become a hot topic in multiple disciplines^13,15,16^, e.g., demography, economics, sociology, education, management, geography. Furthermore, studies on this phenomenon have also paid attention to the continuous integration, intersection, and penetration between various fields, and scholars have reached a consensus that talent mobility plays a key role in science and innovation^1^. However, talents’ spatial mobility involves a multi-aspect, complex, and dynamic process. Early studies on this topic were mostly limited to the static description of spatial phenomena, lacking dynamic analyses from the perspective of mobility. At the end of the twentieth century, complex network theories based on mathematical graph theories and statistical physics began shine light on new methodologies and theoretical support to explore the complexity of talent flow network systems^17,18^.

As one of the regions with the most active economic development, the highest degree of economic openness and the strongest innovation capability in China, the Yangtze River Delta region (“Yangtze River Delta”) is also one of the areas with the most active talents in China. According to the Talent Attraction Ranking of Chinese Cities: 2021, the “magnetic field” effect of the Yangtze River Delta on talents is remarkable: Shanghai, Hangzhou, Nanjing, Suzhou, Ningbo, and similar central cities are among the top ten Chinese cities in talent attraction in 2020. From 2016 to 2020, the proportion of net in-flow of talents to the Yangtze River Delta went up from 4.7 to 6.4%, and the rate has been steadily increasing year by year, reaching higher percentages than other Chinese urban agglomerations^19^. As the only world-class urban agglomeration currently in China, the Yangtze River Delta is extremely significance to the study of highly educated talents and the factors behind this phenomenon in China. Based on data related to the flow of highly educated talents in China and from the perspective of complex networks, this study attempts to reveal the general law of talents’ mobility by exploring the spatial characteristics and influencing factors of the flow network of these talents particularly in the Yangtze River Delta from both national and local perspectives.

## Theoretical framework and research hypotheses

**Figure 1:**
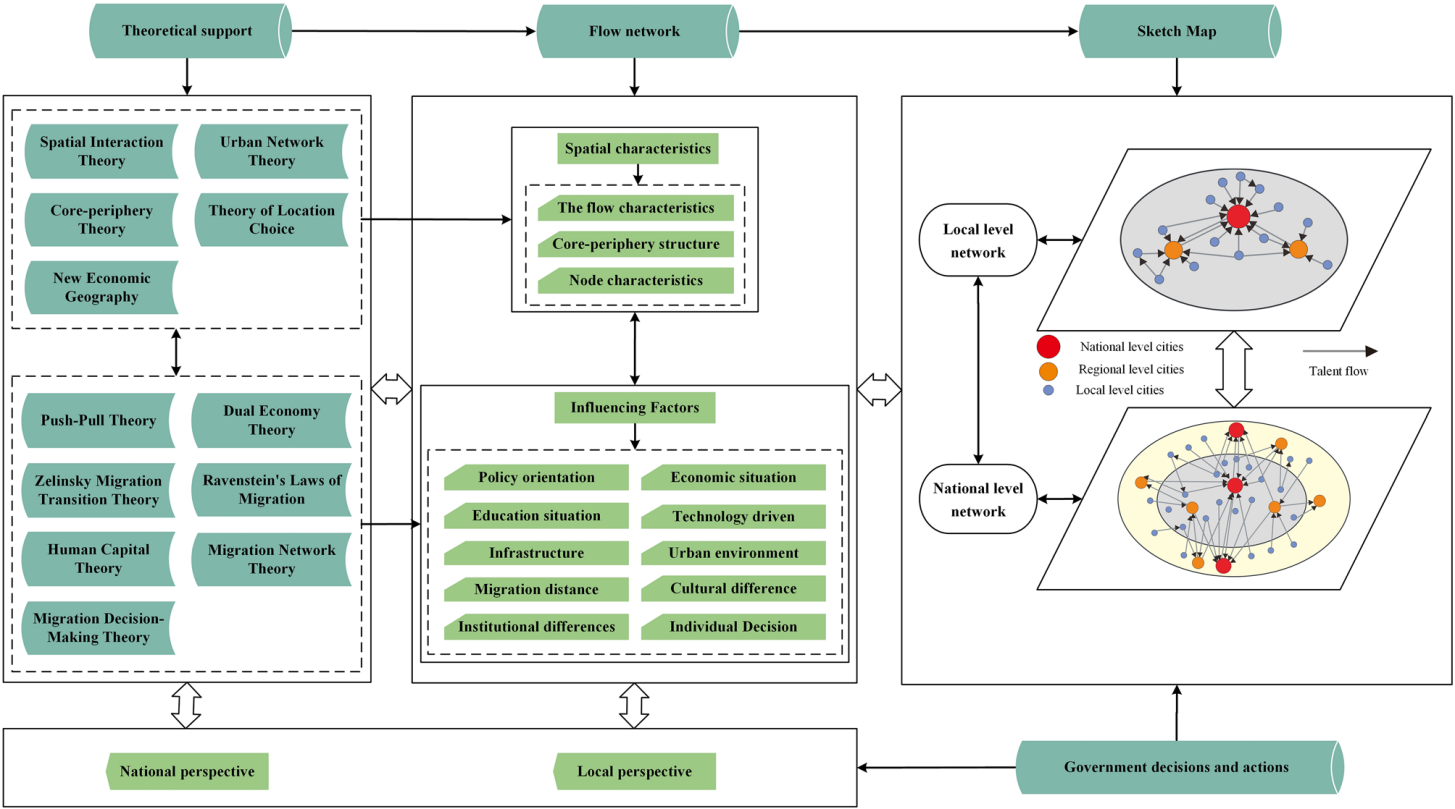
Theoretical framework of the flow network of highly educated talents in the Yangtze River Delta.

Highly educated talents refer to individuals who have received systematic training in scientific and cultural knowledge at institutions of higher education, possessing proficient skills or professional abilities. These talents are important components of high-quality labor force. The spatial differentiation of highly educated talents has become an undeniable fact. New Economic Geography Theory, Talent Location Choice Theory, Core-periphery Theory, Urban Network Theory, and Spatial Interaction Theory, among other established frameworks, provide a theoretical underpinning for better understanding the spatial mobility of highly educated talents (Fig. 1). Highly educated talents exhibit a pronounced characteristic of mobility. Location Choice theory elucidates the spatial selection of work and residential locations by highly educated talents. This theory posits that due to factors such as industrial agglomeration effects, infrastructure conditions, quality of life, and policy support, certain regions or cities are capable of attracting and retaining high-quality talent. Meanwhile, New Economic Geography Theory emphasizes the decisive role of agglomeration economies in the spatial mobility of highly educated talents. Agglomeration economies enable large cities or specific regions to attract a substantial number of talents, as these areas typically offer higher wages, more job opportunities, richer innovation resources, and better living facilities. Spatial Interaction Theory explicates the clustering and diffusion characteristics inherent in the mobility of highly educated talents. Talents interact among different cities or regions, with a tendency to migrate towards 'core' areas that boast greater economic vitality, better job prospects, and enhanced quality of life^20^. The spatial flow of highly educated talents entails two conditions: complementarity and accessibility between regions ^21,22^. In other words, a supply and demand relationship indeed exists combined with the possibility of flow between regions for highly educated talents. The flow of such talents between regions depends on a series of factors in their immigration and emigration places, especially economic development level, cultural education standards, public services, social and cultural atmosphere, and environmental comfort degree^23,24^. In addition, factors like geographic distance, transportation, the attributes of transmission objects, and political, cultural, and social factors between regions can also considerably affect the accessibility between regions for highly educated migrants^25^. According to most recent studies on the flow of high-quality talents, geographic distance impedes the mobility of individuals^5,26,27^, while cultural, institutional, and social proximity tends to facilitate the exchange and cooperation of talents^28–30^. Urban Network Theory offers a framework for more effectively analyzing and explaining the laws governing talent migration between cities. According to this theory, cities do not exist in isolation; instead, each city functions as a node within a network, with talents flowing between these nodes, thereby forming a dynamic network system. In the urban network, there exists a core-periphery structure where core cities possess stronger attraction for talent, while peripheral cities may face pressures of talent outflow. In summary, studying the spatial characteristics of highly educated talents from the perspective of networks is both theoretically and practically necessary. Nowadays, the overwhelming majority of research on talents’ flow draws on the theories and methodologies of social network, so as to reveal the node attribute characteristics, spatial differentiation, topological structure, and hierarchical structure features of talent flow networks^31–34^.

Furthermore, the vast amount of theories and empirical experience accumulated in the research of labor migration and mobility provide a reference for exploring the influencing factors behind the way these highly educated talents move from one region to the other and how these factors affect their mobility flow. Such theories include Ravenstein’s Migration Law, Push–Pull Theory, Zelinsky’s Migration Hypothesis, Dual Labor Market Theory, Human Capital Accumulation Theory, Migration Network Theory, Migration Decision-Making Theory and so on. Scholars have proposed that factors that may influence labor mobility mainly include individual factors, family-related issues, economic prospects, educational costs, environmental comfort, intermediate factors of mobility, and national policies. The boundaries between these factors are still blurred, but scholars suggest that they intertwine and integrate with each other^31,35–37^. Push–Pull Theory holds that population migration is affected by many factors, which "push" immigrants out of a certain area or "pull" immigrants into another area^38^. The “pull” factors include job opportunities, better living conditions, medical conditions, and political freedom. Among them, economic factors are the main reason for population migration. For instance, the migration theory proposed by Ravenstein suggests that people in rural areas migrate more frequently than those in cities and towns, usually looking for more economically developed commercial and industrial centers^39^. Frey’s “migration gateway city”^40^ and Sassen’s “global city hypothesis”^41^ also demonstrate a close relationship between labor migration and economically dynamic big cities. Furthermore, numerous empirical studies have put forward important facts to interpret phenomena related to migration flow. Verginer and Riccaboni revealed that North America and Western Europe are the main agglomerations of various types of talents across the world^1^ and Chinese top talents are widely recognized for their trend to migrate to coastal cities in the southeast^16,31^, following a pattern of labor mobility that mainly flows from the economically underdeveloped areas to economically developed ones. According to Hamilton et al., some “push” factors may include rapid population growth, poverty, political repression, war, and environmental crises associated with resource depletion^42^. Similarly, the migration transition hypothesis proposed by Zelinsky maintains that in the periods of developed post-industrial society and highly developed society^43^, the population migration between cities and within urban agglomerations, as well as the circular migration of highly skilled immigrants and talents between regions, are mainly driven by economic or comfort factors^44^. In this context, environmental comfort can compensate for the lack of economic opportunities, thus becoming an important variable for cities to attract talents^45,46^. Recent studies also consider that air pollution has also become a major impact factor for population migration between Chinese cities, which boosts the likelihood of in-country migration^47^. Mainly from an economic point of view, the Dual Labor Market Theory maintains that labor mobility is conducive to narrowing the gap between urban and rural areas. This theory considers that what drives migration is not the “push factors” in the emigration place, but the “pull factors” of the immigration place, especially in dual labor markets in developed regions^48^. Sanchez-Moral et al. adds that job opportunities, especially those related to workers’ social networks, become the most influential attracting factor of the place of immigration^49^. Human Capital Theory is based on the idea that knowledge, skills and displayed labor ability of workers are the main driving force of modern economic growth^50^. The human capital accumulation and growth model divides each person's fixed time into two parts: one designed for production, and the other designed for education, i.e., for human capital investment^51,52^. From this perspective, the developed regions have more channels to receive professional knowledge training, as well as more opportunities to receive general knowledge. Immigrational flow from less developed regions to more developed regions can be explained as the pursuit of accumulation of human capital by the labor force. Education has become the most important investment in human capital, and this is particularly evident in the process of high-level talents’ flow. For example, when overseas high-level young talents return to China, some factors they consider when selecting their destinations include regional R&D investment, technology system level, university endowment, and academic opportunities^53^. Currently, the improvement of personal scientific research abilities and the sustainable development of careers are the most important factors affecting the flow of top research talents in China^54^. The Immigration Network Theory that emerged in the 1980s provided a brand-new perspective and methodology for labor migration^55^. The theory places labor migration in a research paradigm of "migration system" that mainly examines migration from the perspective of cultural, economic, political, and social connections between emigration and immigration places. Goss and Lindquist explain that like a “migration chain”, labor migration networks connect the current immigrants, the previous ones, and non-immigrants between the origin and the destination place^36^. The networks that incorporate kinships and camaraderie or other relationships based on shared cultures or ethnicity feelings could reduce the costs and risks of migration and boost the sense of security and belonging of migrants, thus playing an important role in maintaining continuous migrations^56–58^. Individual migration decision-making theory examines and explains why and how individuals or households make decisions to move from one location to another. This theoretical framework typically takes into account a range of economic, social, cultural, political, and personal factors influencing migratory behavior, encompassing but not limited to employment prospects, income levels, quality of life, educational resources, family needs, policy environment, and network effects.

As aforementioned, the scholarly world has already come up with sustainable theories on population mobility and sufficient empirical research on talents flow. Existing theories and empirical evidence on labor migration and mobility reveal commonalities and particularities in the spatial distribution and decision-making processes of labor across different tiers, such as economic opportunities and income disparities, educational resources and research environments, policy influences, social network effects, quality of life and livability, push and pull factors, and returns on human capital investment. By integrating classical theories of labor migration with the specific features of modern talent mobility, scholars can better analyze the driving mechanisms behind the flow of highly educated talents. While existing studies have offered many enlightening perspectives on talent mobility, they often concentrate on single or a select few influencing factors. So, synthesizing multiple factors to develop a more holistic theoretical model that accurately forecasts and directs real-world talent flow trends remains a formidable challenge. Therefore, this study endeavors to examine the influencing factors of the flow network for highly educated talents in the Yangtze River Delta region from national and local perspective. Based on the above-mentioned models and theories related to talent migration, and referring to the conclusions of previous studies on the driving mechanisms of talents' flow, this research explores the influencing factors on the flow network of highly educated talents in the Yangtze River Delta in terms of regional economic development, education level, scientific and technological level, infrastructure conditions, regional environmental conditions, and geographical distances of talents’ flow, as well as cultural identity, institutional difference, social form gaps, and other impacting dimensions related to migration. After considering all these factors, the following hypotheses are proposed:

### Hypothesis 1

Regional economic development affects the flow of highly educated talents. In in-flow cities, higher economic levels are associated with more intense immigration flows of highly educated talents; in out-flow cities, lower economic levels are associated with more emigrations of highly educated talents.

### Hypothesis 2

The level of education development affects the flow of highly educated talents across the Yangtze River Delta. The degree, scale, and quality of education in in-flow cities positively impact the flow of highly educated talents.

### Hypothesis 3

The level of scientific and technological development affects the flow of highly educated talents across the Yangtze River Delta. High-tech enterprises in destination cities exert a positive influence on the flow of highly educated talents.

### Hypothesis 4

Infrastructure conditions influence the flow of highly educated talents across the Yangtze River Delta. The better the infrastructure of in-flow cities, the stronger their attraction for highly educated talents.

### Hypothesis 5

The urban environment affects the flow of highly educated talents across the Yangtze River Delta. Serious pollution issues in out-flow cities are likely associated with more out-flows of highly educated talents; in in-flow cities, pleases environments are associated with the incoming flow of highly educated talents.

### Hypothesis 6

Geographical distance negatively impacts the flow of highly educated talents across the Yangtze River Delta. Longer migration distances between two cities are associated with weak migration flow.

### Hypothesis 7

The smaller the differences in culture, institution, and social form between the emigration and immigrant cities, the stronger the flow of highly educated talents between these cities.

## Data and methodology

### The study area

**Figure 2:**
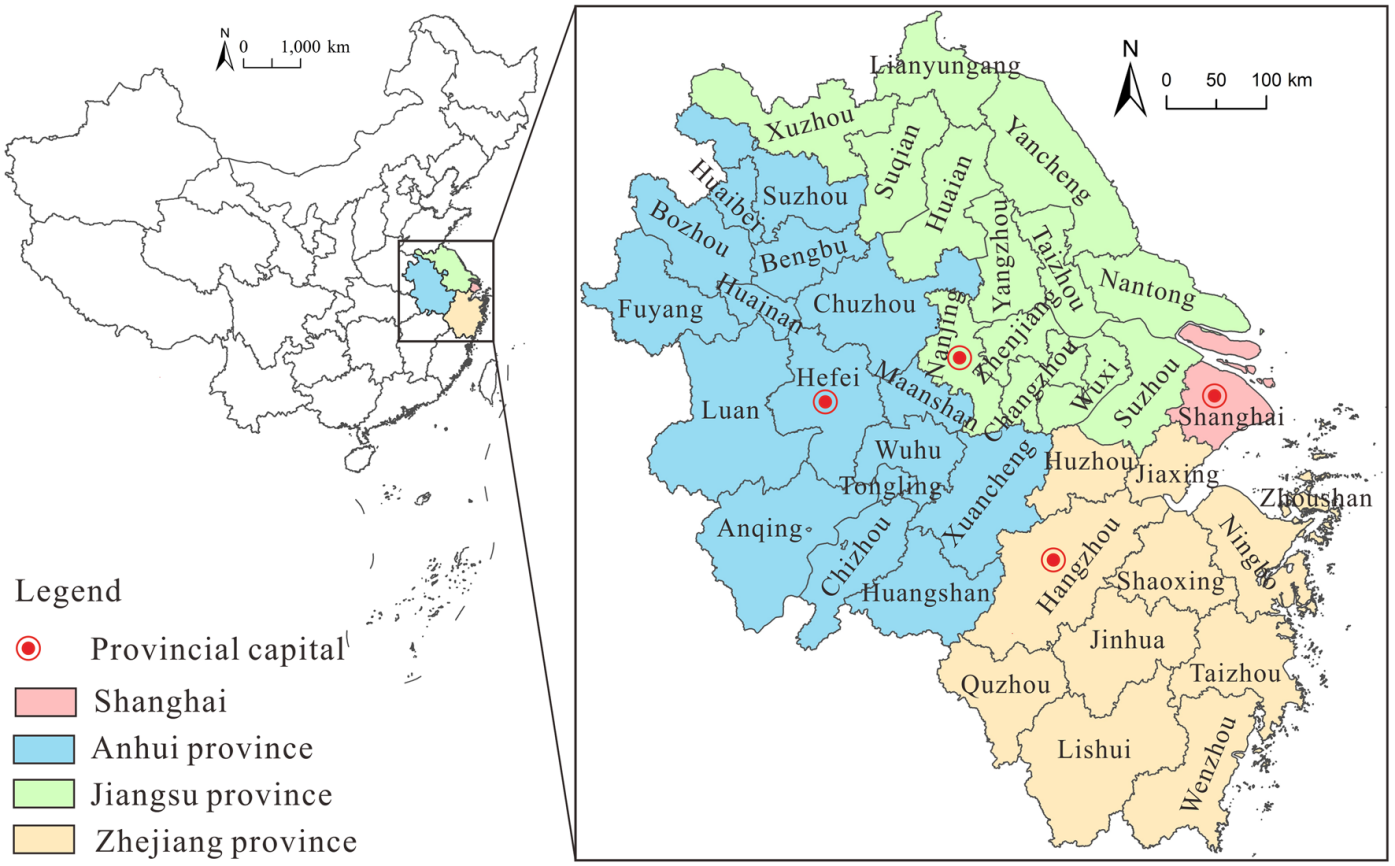
Location map of the Yangtze River Delta region. *Source*: Based on the standard map service website of the Ministry of Natural Resources, the approval number is GS (2020)4630 and GS (2020)3189. The base map boundary is not modified.

According to the “Outline of the Regional Integrated Development Plan of the Yangtze River Delta” approved by the State Council of China in 2019, the Yangtze River Delta region includes three provinces and one city, i.e., Shanghai, Jiangsu Province, Zhejiang Province, and Anhui Province. It is composed of 41 cities above the prefecture level. For the comprehensive production of all maps in this study, we relied upon ArcGIS 10.8 software, drawing upon the standard map services sourced from the official website of the Ministry of Natural Resources. It is important to emphasize that the original base map boundaries remained unaltered throughout the course of our analysis. Geo-location and composition of the Yangtze River Delta region are shown in Fig. 2.

### Data

Highly educated talents included in this study are those with a bachelor’s degree or higher, i.e., talents who hold a bachelor, master, or doctoral degree. The data on the flow of highly educated talents in the Yangtze River Delta stem from the 2017 China Migrants Dynamic Survey (CMDS) released by the National Health Commission of China. This dataset is from a large-scale, nationwide sampling survey of migrating population conducted by the National Health Commission. The survey utilized the data on the entire migrating population from 2016 as its sampling frame, adopting a multi-stage, stratified probability sampling method that is proportionate to the size of the migrating population. It covers areas with high concentrations of incoming migrating populations across all 31 provincial-level administrative units in China (excluding Hong Kong, Macao, and Taiwan). The sample size is nearly 200,000 households, and the subjects of the survey are individuals aged 15 years or older who have been residing in the destination area for one month or longer and do not hold local household registration status. The content of the survey encompasses basic demographic information of migrant populations and their family members, the scope and trends of migration, employment and social security, income and expenditure, housing conditions, basic public health services, marriage, birth control and family planning service management, children’s migration and education, as well as psychological and cultural aspects. This study acquires information on the educational background of the surveyed, the out-flow, and the in-flow regions. Taking the administrative cities at the prefecture level or above in China as the primary research unit, with the highly educated talents flowing between the cities as linkages, a new flow network of highly educated talents is proposed, categorizing the highly educated talents across the Yangtze River Delta as part of a directed complex network. At the national scale, the flow network of highly educated talents between the Yangtze River Delta and the whole nation involves 244 cities; at the local scale, the flow network of highly educated talents in the Yangtze River Delta involves 41 cities.

### Research methodologies

#### Network feature measurement model

Graph Density (Dg) refers to the tightness of the connections between nodes in a network. In the analysis of social networks, Graph Density is used to summarize the total distribution of individual connections to measure the gap between this distribution and a whole network. The more connections between network nodes, the greater the Graph Density. The Graph Density of a directed relational network can be expressed by the ratio of the number of the actually possessed relations to the total number of theoretically possible maximum relations:

1 $${D}_{g}=\frac{m}{n\left(n-1\right)}$$

where *Dg* indicates the Graph Density; *m* indicates the number of actually owned connections in the network; *n* refers to the number of network nodes. The value range of the Graph Density is 0–1; when a network is fully connected internally, its Graph Density is 1.

Degree Centrality refers to the number of other nodes directly connected to a node, representing the degree of connection. If a node is directly connected to many others, it has high degree centrality. For example, in the network of talent flow in the Yangtze River Delta, the degree centrality of a node represents the number of cities that have a relationship with a certain city in term of flow of these talents. The greater the degree centrality, the more cities that have a relationship with the certain city in terms of flow of highly educated talents. In a directed relational network, the degree centrality can be divided into out-degree and in-degree centrality. The measurement model of degree centrality is as follows:

2 $$ C_{d} \left( i \right) = \sum\limits_{{j = 1}}^{n} {a_{{ij}} }  $$

where ${C}_{d}\left(i\right)$ represents the degree centrality of a city; ${a}_{ij}$ represents the urban flow matrix of highly educated talents’ flow networks, with a value of 1 for those with a flow relationship and 0 for those without a flow relationship.

Weighted Degree Centrality is the weight of an edge directly connected to a node. In a directed relational network, this measurement factor can be divided into weighted out-degree centrality and weighted in-degree centrality. In the flow network of highly educated talents in the Yangtze River Delta, the weighted degree centrality of nodes indicates the sum of the highly educated talents flowing into and out of a city. The measurement model of weighted degree centrality is as follows:

3 $$ C_{w} \left( i \right) = \sum\limits_{{j \in v}} {w_{{ij}} }  $$

where ${C}_{w}\left(i\right)$ indicates the weighted degree centrality of a city; *V* represents the set of nodes that are directly connected to node *i*; *w*_*ij*_ represents the number of highly educated talents flowing between cities *i* and *j*. Cities with higher weighted degree centrality play a more important role in the flow network of highly educated talents in the Yangtze River Delta.

#### Model of negative binomial regression

As shown in Table 1, the number of highly educated talent flows between cities is count data consisting of non-negative integers, which meets the requirements for the dependent variable in the negative binomial regression model. Moreover, the variance of the dependent variable is greater than its mean, demonstrating an overdispersion characteristic in the data, and the negative binomial regression model is adept at handling such data that exhibits a distribution pattern more dispersed than a simple Poisson distribution, thereby allowing for a better capture of the variability within the data. The presence of clustering effects or overdispersion among the observational data indicates that the different observation outcomes are not completely independent from one another. The negative binomial regression model is able to adapt to such data structures where it takes into account the interdependence or clustering among the observational data in the distribution of the dependent variable. In summary, under this research scenario, the negative binomial regression model is better suited to accurately describe and predict the number of highly educated talent flows between cities, as well as the relationships between these flows and various independent variables. Its formula is as follows:

4 $$ \begin{aligned} N_{ij} = & \alpha + \beta_{1} GDP_{i} + \beta_{2} GDP_{j} + \beta_{3} Colleges_{j} + \beta_{4} Universities_{j} + \beta_{5} Science_{j} + \beta_{6} Hospitals_{j} \\ & + \beta_{7} Buses_{j} + \beta_{8} Greenbelt_{j} + \beta_{9} Pollution_{i} + \beta_{10} Distance_{ij} + \beta_{11} Province_{ij} + \varepsilon_{i} \\ \end{aligned} $$

where the dependent variable *N*_*ij*_ indicates the number of highly educated talents flowing from city *i* to city *j*, and it is the explained variable; *α* is a constant term; *β*_*1-11*_ is the coefficient to be estimated; and *ε*_*i*_ is the random error term. Among the explanatory variables, in terms of regional economic development, *GDP*_*i*_ is the gross regional product of out-flow city *i*, and *GDP*_*j*_ is the gross regional product of in-flow city *j*. In terms of the development level of science and education, given the scale and level of education of each region, the number of higher education institutions in an in-flow city and whether it has double-first-class universities are used as core variables. *Colleges*_*j*_ is the number of colleges and universities in in-flow city *j*; *Universities*_*j*_ is a dummy variable, indicating whether there are any double-first-class universities in in-flow city *j*. If yes, then the value of 1 will be assigned; otherwise, 0 is assigned. The development level of science and technology is represented by the number of high-tech enterprises (*Science*_*j*_) in each city. Since there is currently no authoritative statistical yearbook available for the high-tech enterprise situations across all cities, this study acquires related data on high-tech enterprises through searching the open data platform (Qcc.com, https://www.qcc.com/). Although Qcc.com is a commercial open data platform, its data originates from the National Enterprise Credit Information Publicity System, and has been widely utilized in numerous research studies, thus rendering the data highly credible. Regarding infrastructure conditions, the numbers of hospitals and buses in a destination city are selected as the core variables: *Hospitals*_*j*_ is the number of hospitals in in-flow city *j*, and *Buses*_*j*_ is the number of buses circulating in the city. In terms of regional environmental conditions, both positive and negative indicators are considered. The green coverage rate of the built-up areas in in-flow cities and the PM 2.5 emission in out-flow cities are selected as core variables: *Greenbelt*_*j*_ is the green coverage rate of the built-up areas in in-flow city* j*, and *Pollution*_*i*_ is the PM2.5 pollutant amount in out-flow city *i*. The accessibility of highly educated talents in their migration flow is subject to obstacles aspects like geographical distance, transportation, institution, culture, and society. *Distance*_*ij*_ is the geographical distance between out-flow city *i* and in-flow city *j*. In addition, the physical distance between cities was calculated based on their longitudes and latitudes. *Province*_*ij*_ is a dummy variable, indicating whether the out-flow city *i* and the in-flow city *j* belong to the same province: if so, the value of 1 is assigned; otherwise, 0 is assigned. This variable reflects the proximity in institution, culture, society and other aspects affecting the migration flow of highly educated talents.

**Table 1:** Descriptive statistics and data sources.

Variables	Observations		Mean value		Standard deviation		Maximum		Minimum		Data sources
	National	Local	National	Local	National	Local	National	Local	National	Local	
*N*_*ij*_	667	283	2.02	4.52	2.41	7.18	20.00	68.00	1.00	1.00	National Health Commission of the People's Republic of China
*GDP*_*i*_	667	283	30,502,582.83	28,180,277.99	58,534,449.36	50,537,220.75	306,329,900.00	306,329,900.00	349,527.00	3,238,217.00	China city statistical yearbook 2017
*GDP*_*j*_	667	283	144,220,247.26	83,283,687.02	117,962,290.07	96,076,237.92	306,329,900.00	306,329,900.00	1,093,212.00	2,869,916.00	China city statistical yearbook 2017
*Colleges*_*j*_	667	283	39.58	24.10	26.39	22.14	91.00	64.00	0.00	1.00	Educational Statistics Yearbook of China 2017
*Universities*_*j*_	667	283	0.44	0.24	0.50	0.43	1.00	1.00	0.00	0.00	Educational Statistics Yearbook of China 2017
*Science*_*j*_	667	283	13,180.34	8505.51	9720.68	8025.65	28,421.00	24,747.00	48.00	277.00	High-tech Enterprises of Enterprise Business Information Query System(qcc.com)
*Hospitals*_*j*_	667	283	402.35	283.24	243.76	174.21	1606.00	656.00	9.00	62.00	China city statistical yearbook 2017
*Buses*_*j*_	667	283	9549.76	5381.92	7397.01	5351.35	33,325.00	16,693.00	159.00	183.00	China city statistical yearbook 2017
*Greenbelt*_*j*_	667	283	41.67	41.88	5.84	2.46	61.58	48.69	3.07	36.94	China city statistical yearbook 2017
*Pollution*_*i*_	667	283	41.48	45.04	15.89	10.15	78.93	59.49	6.80	21.42	China city statistical yearbook 2017
*Distance*_*ij*_	667	283	1,052,222.21	201,557.31	476,320.31	138,493.43	3,425,682.05	663,755.74	120,741.62	29,590.94	Measured based on the cities’ latitude and longitude
*Province*_*ij*_	667	283	0.00	0.55	0.00	0.50	0.00	1.00	0.00	0.00	Measured by whether the city belongs to the same province

## The spatial pattern of the inter-city flow network of highly educated talents between the Yangtze River Delta and the whole nation

### The spatial characteristics of inter-city correlation from a national perspective

The inter-city flow network of highly educated talents between the Yangtze River Delta and the whole nation demonstrates evident spatial heterogeneity and hierarchical levels. In terms of spatial distribution, from a national perspective, the flow of highly educated talents in the Yangtze River Delta is deeply rooted in the central and eastern regions of China. With cities in the Yangtze River Delta as the source, Beijing-Tianjin-Hebei urban agglomeration, Pearl River Delta, Northeast, central, and Chengdu-Chongqing urban agglomeration are the main connection areas for the flow of highly educated talents in the Yangtze River Delta.

As shown in the flow trends, the flow to large cities and geographically adjacent cities is the major rule for the inter-city flow of highly educated talents between cities in the Yangtze River Delta and the whole country. The flow of highly educated talents from outside the region to big cities inside the region constitutes the backbone of the inter-city flow network. Shanghai, Hangzhou, Suzhou, and Nanjing are important cities involved in the inter-city flow network of highly educated talents between the Yangtze River Delta and the whole nation, forming the main in-flow regions. On the other hand, cities in Shandong, Henan, Hubei, Jiangxi, and Fujian around the Yangtze River Delta region are the main sources of highly educated talents in the area.

**Figure 3:**
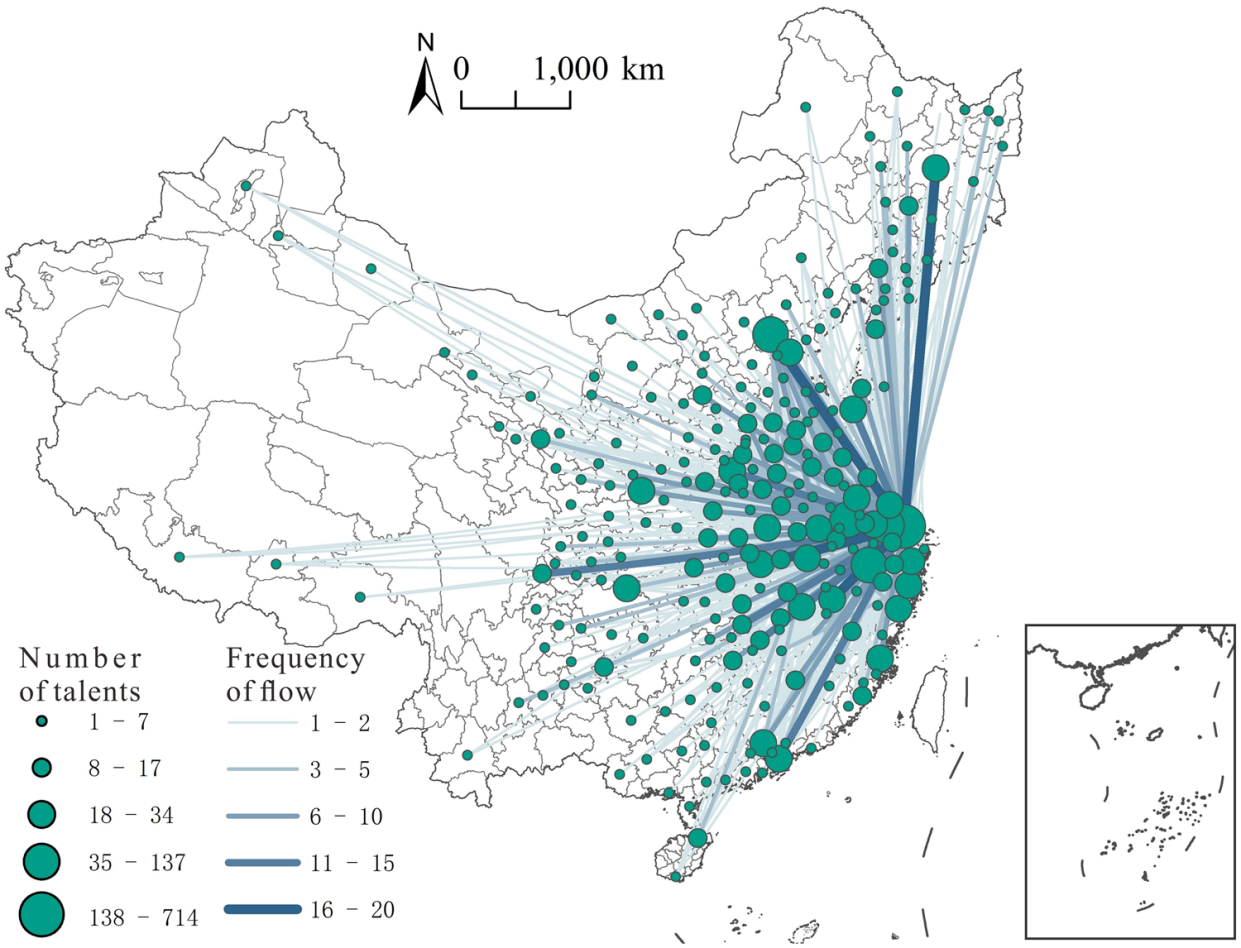
Spatial pattern of the inter-city flow network of highly educated talents between the Yangtze River Delta and the whole nation. *Source*: Based on the standard map service website of the Ministry of Natural Resources, the approval number is GS (2020)4630. The base map boundary is not modified.

From a national perspective, the inter-city flow network of highly educated talents between the Yangtze River Delta and the whole nation has formed a spatial pattern of convergence in the region and a pattern of divergence across the country (Fig. 3). The number of highly educated talents flowing into the Yangtze River Delta region is far greater than that flowing out, which means that it has a strong capacity to attract highly educated talents from across the country.

The frequency of highly educated talents flowing into the region from outside is 1032, accounting for 76.05% of the total frequency. From a national perspective, Shanghai is the main destination for highly educated talents, with 589 people flowing to Shanghai, accounting for 43.40% of the total in-flow frequency. Furthermore, the top ten cities in the degree centrality ranking for the flow network of highly educated talents between the Yangtze River Delta and the whole nation are mainly the cities within the region, and the in-flow degree of these cities is generally higher than their out-flow degree, with a positive net in-flow degree.

In addition, it can be observed that the Yangtze River Delta is an important in-flow destination for highly educated talents across the country: the talents across the nation converge to the Yangtze River Delta, especially in its core cities. For example, Shanghai, Hangzhou, Suzhou, and Nanjing are important in-flow cities, but large cities outside the region also have some attractive factors for the highly educated talents of the Yangtze River Delta. For example, Beijing, Tianjin, and Shenzhen have higher in-flow degrees than out-flow ones, with a positive net in-flow degree. However, common cities outside the region have weak capacities to attract highly educated talents from the Yangtze River Delta, so their in-flow degrees are generally lower than the out-flow ones. As a result, these regions also have a negative net in-flow degree, indicating that the highly educated talents in the Yangtze River Delta mainly flow to first-tier cities in China.

**Figure 4:**
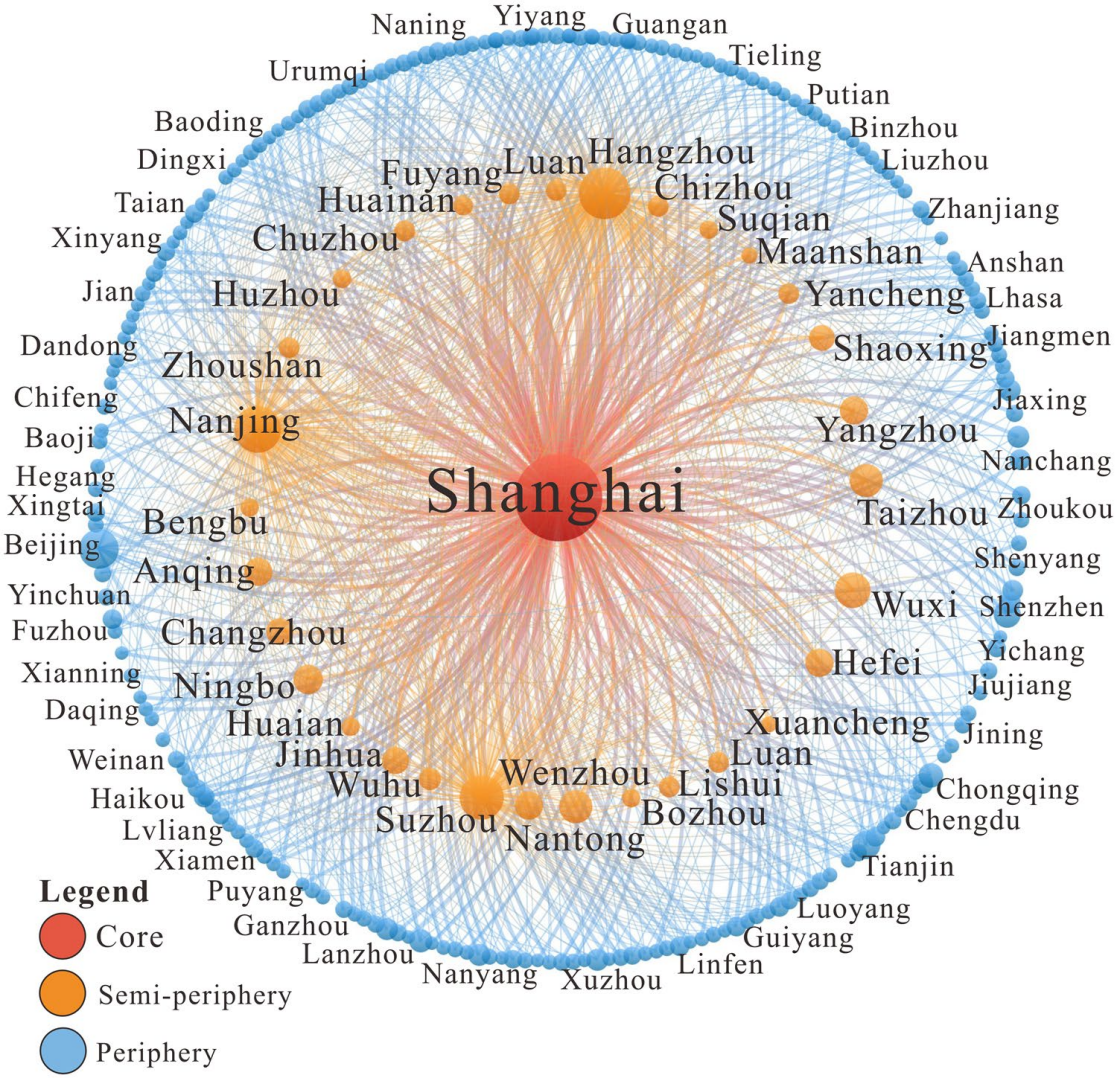
Core-periphery structure of the inter-city flow network of highly educated talents between the Yangtze River Delta and the whole nation.

By using the Hierarchical Clustering method in the Pajek block model analysis, the hierarchical division were obtained based on the weighted degree centrality, and it was possible to divide the inter-city flow network of highly educated talents between the Yangtze River Delta and the whole nation into three levels. The division showed that this network presents a “pyramid-like” hierarchical structure, with a noticeable “core-periphery” hierarchical progressive form, which can be further divided into three major urban groups: (i) core area, (ii) semi-periphery area, and (iii) periphery area (Fig. 4). The size of the nodes shown in the figure is proportional to the weighted degree centrality of a city, and the size of edges is positively related to the number of highly educated talents flowing between two cities.

As shown in Fig. 4, Shanghai is the only core city in the inter-city flow network of highly educated talents between the Yangtze River Delta and the whole nation, and it is located at the center of the map. The average degree centrality, average intensity centrality, average closeness centrality, average betweenness centrality, density, and other network statistical indicators of cities in the core area are much higher than the average measures of the entire network (Table 2). As shown, 81.56% of the cities in the network have connections with Shanghai, which has a weighted in-degree centrality of 664, accounting for 48.93% of the total. This shows that on the national scale, Shanghai has, as the top city in the Yangtze River Delta region, attracted nearly half of the highly educated talents of the inter-city flow network of highly educated talents between the Yangtze River Delta and the whole nation. Because it connects a lot of other cities, Shanghai became the hub node of the network and an important distribution center for highly educated talents across the country.

**Table 2:** Characteristics of the hierarchical structure of the inter-city flow network of highly educated talents between the Yangtze River Delta and the whole nation.

Level	No. nodes	Average degree	Average weighted degree	Average closeness centrality	Average betweenness centrality	Density
Core	1	190.00	714.00	0.3650	10,374.13	0.8589
Semi-periphery	31	14.52	19.71	0.2887	485.59	0.4569
Periphery	212	3.29	5.55	0.2842	74.81	0.0001
Whole network	244	5.48	11.12	0.2850	169.21	0.0099

There are 31 cities in the semi-periphery area, which are all inside the Yangtze River Delta region. Among them, 12 cities are in Anhui Province, namely: Hefei, Anqing, Lu'an, Wuhu, Fuyang, Bengbu, Bozhou, Chuzhou, Chizhou, Huainan, Ma'anshan, and Xuancheng; 10 cities are in Jiangsu Province: Suzhou, Nanjing, Wuxi, Yangzhou, Nantong, Changzhou, Lianyungang, Huai'an, Yancheng, and Suqian; and 9 cities are in Zhejiang Province, namely: Hangzhou, Wenzhou, Taizhou, Ningbo, Jinhua, Shaoxing, Huzhou, Zhoushan, and Lishui. Most of the cities in the Yangtze River Delta are located in the semi-periphery area of the flow network of highly educated talents between the Yangtze River Delta and the whole nation, indicating that all the cities inside the region have actively participated in the flow network. The network statistical indicators for this area are also higher than the average of the entire network. The cities in the periphery area have an average weighted in-degree centrality of 319 and an average weighted out-degree centrality of 292, indicating that they are the in-flow cities of highly educated talents across the country and “export” these talents to cities outside the region.

Furthermore, most of the cities are located in the periphery area of the inter-city flow network of highly educated talents between the Yangtze River Delta and the whole nation. There are 212 cities in this area, accounting for 86.86% of the total. These regions play an auxiliary role in the network. The periphery cities are mainly the out-flow places for highly educated talents, and in 182 cities, the out-flow of talents is greater than the in-flow, accounting for 85.85% of the total. In the periphery area, the Graph Density is only 0.0001, the average degree centrality is 3.29, the average weighted degree centrality is 5.55, and the average betweenness degree centrality is 74.81. All these network indicators are lower than the average value of the network, showing that in the inter-city flow network of highly educated talents between the Yangtze River Delta and the whole nation, these cities are less active, and most cities act as talent-exporting regions sending highly educated talents to the core and semi-periphery areas.

### The spatial characteristics of urban centrality from a national perspective

**Figure 5:**
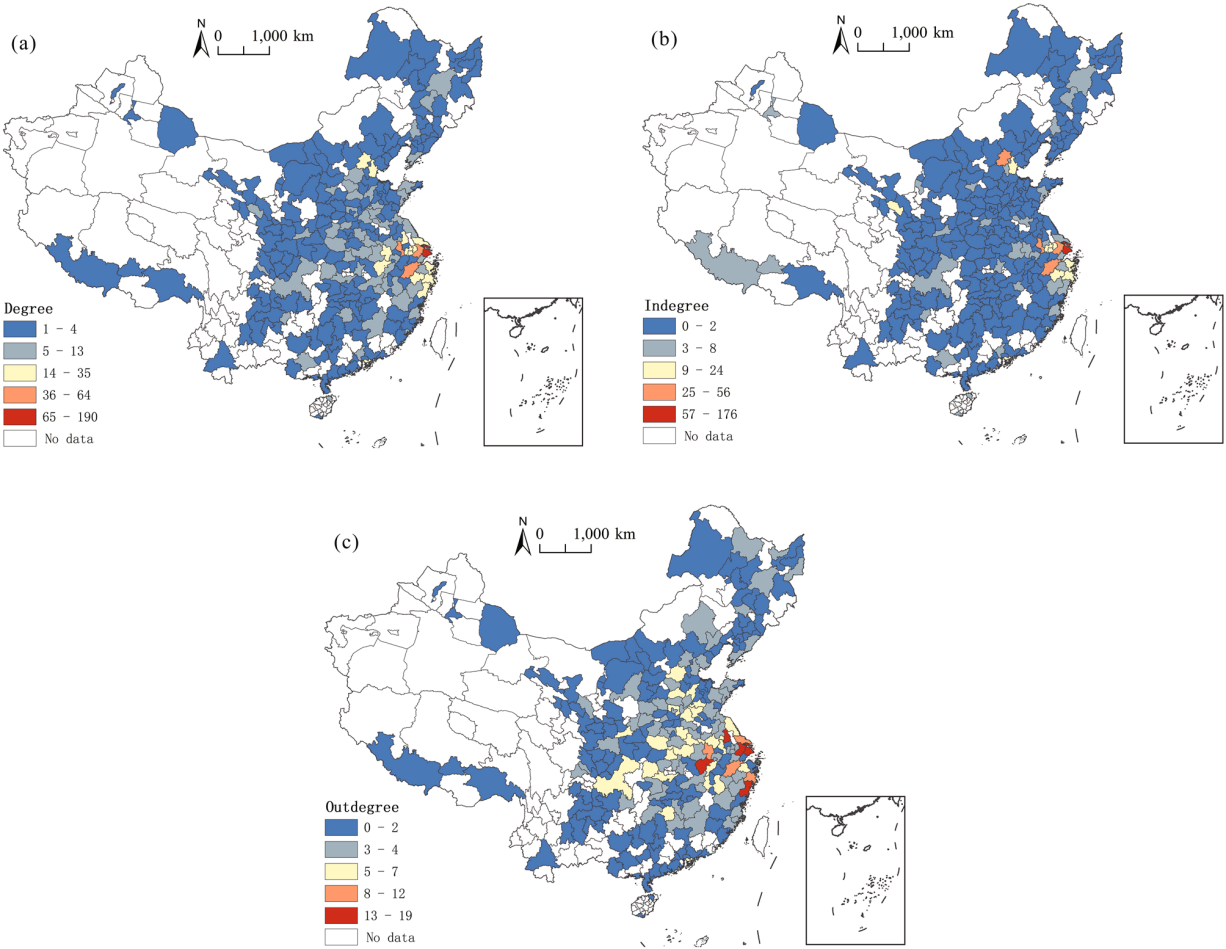
The spatial distribution of degree centrality ((**a**) degree centrality; (**b**) in-degree centrality; (**c**) out-degree centrality) of nodes of the inter-city flow network of highly educated talents between the Yangtze River Delta and the whole nation. *Source*: Based on the standard map service website of the Ministry of Natural Resources, the approval number is GS (2020)4630. The base map boundary is not modified.

The spatial distribution of the degree centrality of the inter-city flow network of highly educated talents between the Yangtze River Delta and the whole nation is uneven, generally showing a spatial pattern of dense eastern parts mixed with sparse western parts (Fig. 5). In terms of degree centrality, cities in the eastern coastal areas generally have a higher value. Especially, the three major urban agglomerations, i.e., the Yangtze River Delta, the Pearl River Delta, and the Beijing-Tianjin-Hebei regions, show considerably high values of degree centrality.

In the Yangtze River Delta region, there are many node cities with a high degree centrality in the network, e.g., Shanghai, Hangzhou, Nanjing, Suzhou, Wuxi, Wenzhou, Taizhou, Hefei, Yangzhou, Ningbo, Nantong, Anqing, Jinhua, Changzhou, and Shaoxing. In addition, some large cities and regional centers outside the Yangtze River Delta region, e.g., Beijing and Tianjin in the Beijing-Tianjin-Hebei region, Shenzhen and Guangzhou in the Pearl River Delta region, Wuhan, Nanchang, and Zhengzhou in the central region, and Chongqing, Xi'an and Lanzhou in the western region, also perform well in terms of degree centrality. From the perspective of in-degree centrality, the spatial distribution of in-degree centrality of nodes of the inter-city flow network of highly educated talents between the Yangtze River Delta and the whole nation is concentrated, especially in the Yangtze River Delta region, indicating that the region is the main in-flow place of highly educated talents across the country. Furthermore, this region shows a spatial pattern based on “one superpower and multiple powerhouses”. Shanghai connects the most cities across the country, with a degree centrality of 176. Such cities as Hangzhou, Nanjing, Suzhou, Wuxi, Taizhou, and Ningbo have high degree centrality, which means they absorb highly educated talents from many cities across the nation. Outside the Yangtze River Delta region, Beijing, Tianjin, and Shenzhen are the main in-flow cities of highly educated talents from the Yangtze River Delta, with high degree centrality. Contrary to the in-degree centrality, the spatial distribution of the out-degree centrality of nodes of the inter-city flow network of highly educated talents between the Yangtze River Delta and the whole nation is relatively even. The cities in the Yangtze River Delta region are all places with high out-degree centrality, indicating that this region provides highly educated talents to the nation while also attracting such talents from the whole country. Wenzhou, Yangzhou, Anqing, Shanghai, Suzhou, and Hefei have a high out-degree centrality, but there is no significant gap between these and other cities. All cities in the country have seen their highly educated talents flow out to the Yangtze River Delta region, without any region having a particularly high degree centrality.

**Figure 6:**
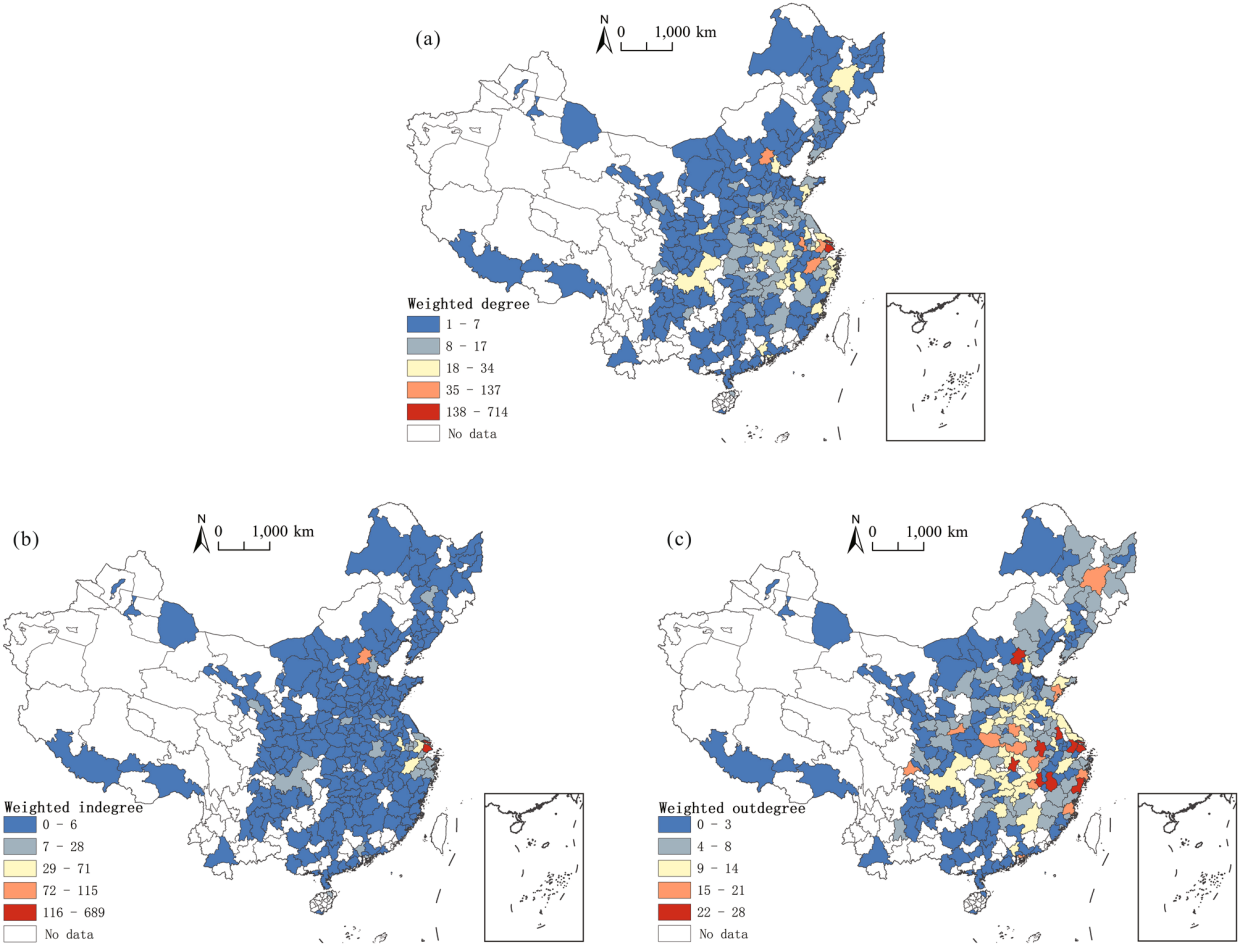
Spatial distribution of the weighted degree centrality ((**a**) weighted degree centrality; (**b**) weighted in-degree centrality; (**c**) weighted out-degree centrality) of nodes of the inter-city flow network of highly educated talents between the Yangtze River Delta and the whole nation. *Source*: Based on the standard map service website of the Ministry of Natural Resources, the approval number is GS (2020)4630. The base map boundary is not modified.

The polarization of the weighted degree centrality of nodes of the inter-city flow network of highly educated talents between the Yangtze River Delta and the whole nation is significant, showcasing a spatial pattern based on “one superpower and many powerhouses” (Fig. 6a). The weighted centrality of Shanghai is 714, which makes it the city with the most frequent flow of talents in the network. Core cities or economically developed cities within the region (such as Hangzhou, Nanjing, Hefei, Suzhou, Wuxi, Taizhou, Ningbo, Jinhua, Changzhou, Xuzhou, Nantong, Shaoxing, Wenzhou, etc.) and some first-tier big cities or provincial capitals outside the region (such as Beijing, Tianjin, Shenzhen, Guangzhou, Lanzhou, Chongqing, Haikou, Zhengzhou, Changchun, Qingdao, Nanchang, Xi'an, Taiyuan, Guiyang, etc.) show relatively high weighted centrality, which means they have actively participated in the flow network of highly educated talents between the Yangtze River Delta and the whole nation.

Shanghai and Beijing are the main destinations for the intra- and extra-regional flows of highly educated talents in China, ranking first and second in terms of weighted in-degree centrality, respectively (Fig. 6b). The weighted in-degree centrality of Shanghai is 689, accounting for 50.77% of the total, indicating that it is the primary destination for the flow of highly educated talents across the country to the Yangtze River Delta region. In addition, Hangzhou, Nanjing, Suzhou, and Wuxi in the Yangtze River Delta region are also strongly attracted to highly educated talents across the country, with high weighted in-degree centrality. However, the primary destination for the flow of highly educated talents in the Yangtze River Delta region to other areas across is Beijing, with a weighted in-degree centrality of 115, indicating that talents are considerably attracted by Beijing’s political and economic advantages. In contrast to the weighted in-degree centrality, the spatial distribution of the weighted out-degree centrality of the inter-city flow network of highly educated talents between the Yangtze River Delta and the whole nation is relatively even, with no obvious polarization (Fig. 6c). The highest value of the weighted out-degree centrality appears in Wenzhou (28), with small gaps compared with that of other cities. The cities in the Yangtze River Delta with a heavy flow of highly educated talents are not the top cities in the region, but the prefecture-level cities around the core regions, such as Wenzhou, Suzhou, Yangzhou, Taizhou, etc., indicating that the flow within the region follows the siphon effect of large cities. The high values of extra-regional weighted out-degree centrality present a “crescent-shaped” distribution around the Yangtze River Delta, indicating that the region is highly attractive to talents in surrounding provinces, such as Shandong, Henan, Hubei, Hunan, Jiangxi, and Fujian. This also indicates that extra-regional flow in the area follows the rule of geographic proximity.

## The spatial pattern of the inter-city flow network of highly educated talents locally in the Yangtze River Delta

### The spatial characteristics of inter-city correlation from a local perspective

**Figure 7:**
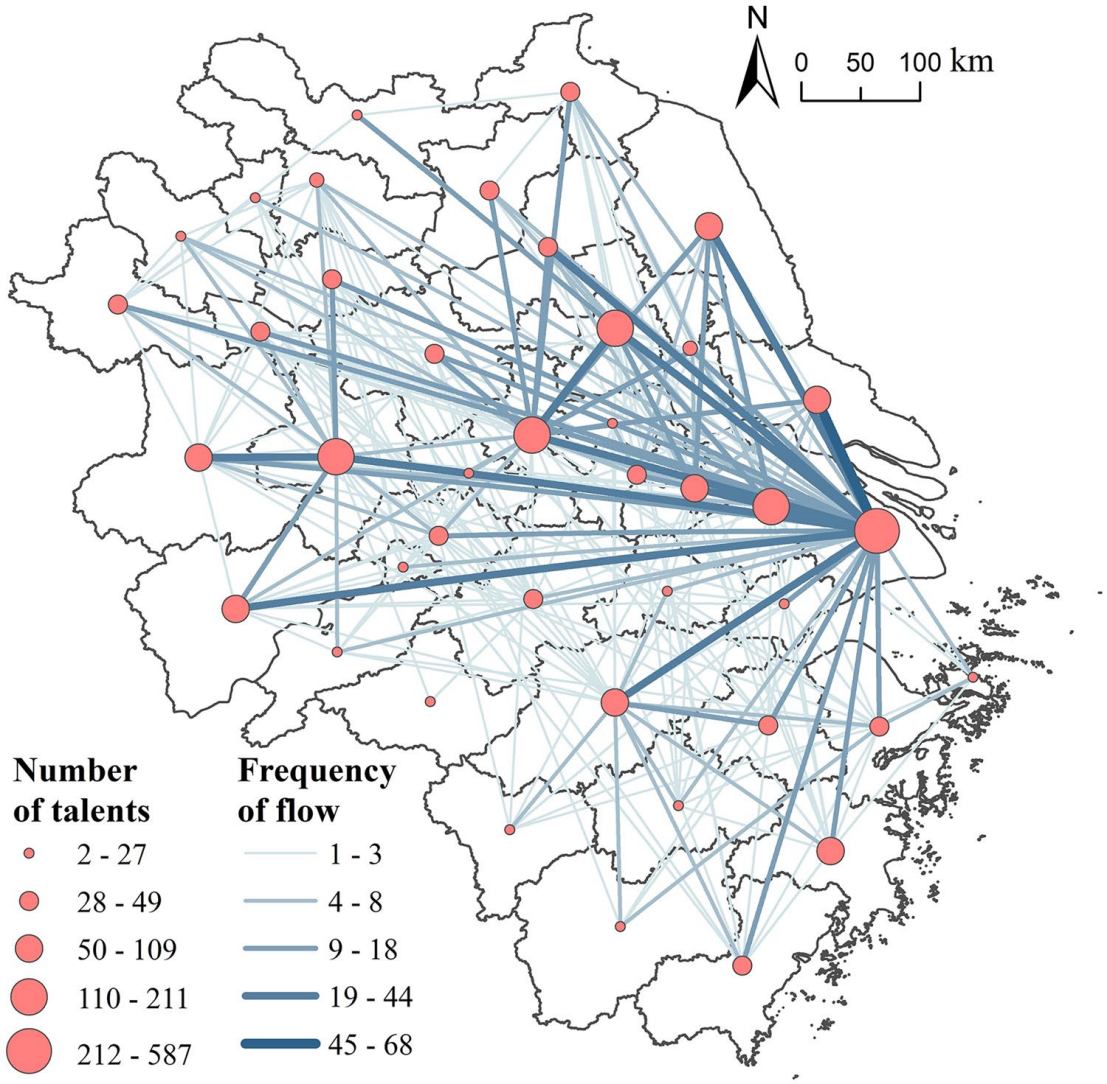
Spatial pattern of the flow network of highly educated talents locally in the Yangtze River Delta. *Source*: Based on the standard map service website of the Ministry of Natural Resources, the approval number is GS (2020)3189. The base map boundary is not modified.

The inter-city flow network of highly educated talents locally in the Yangtze River Delta has tight spatial connections, demonstrating high density and high connectivity (Fig. 7). The network is fully connected, with no isolated nodes and its Graph Density is 0.186. All cities in the region have participated in the flow network of highly educated talents.

From the perspective of spatial structure, the in-flow of highly educated talents to Shanghai from the provincial capital cities with higher administrative levels and the economically developed core cities in the region constitutes the backbone of the inter-city flow network of talents in the Yangtze River Delta. Spatially, a pattern of convergence to Shanghai is identified. Central cities inside the region, especially Shanghai, have a noticeable siphon effect on highly educated talents. A total of 573 highly educated talents have flowed into Shanghai, accounting for 44.80% of the total flow volume. From a local perspective, Nantong, Yangzhou, Yancheng, Suzhou, Hangzhou, Hefei, Anqing, Nanjing, Huai’an, Wuxi, Wenzhou, Shaoxing, and Taizhou are the main sources of highly educated talents flowing to Shanghai. Six cities, including Shanghai, Nanjing, Hefei, Hangzhou, Wuxi, and Xuzhou deliver a positive net in-flow of highly educated talents, with a relatively large in-flow volume, indicating that provincial capital cities and economically developed cities have institutional, economic, and resource advantages that attract these talents. On the other hand, most cities in the region see a negative net in-flow volume of these talents.

**Figure 8:**
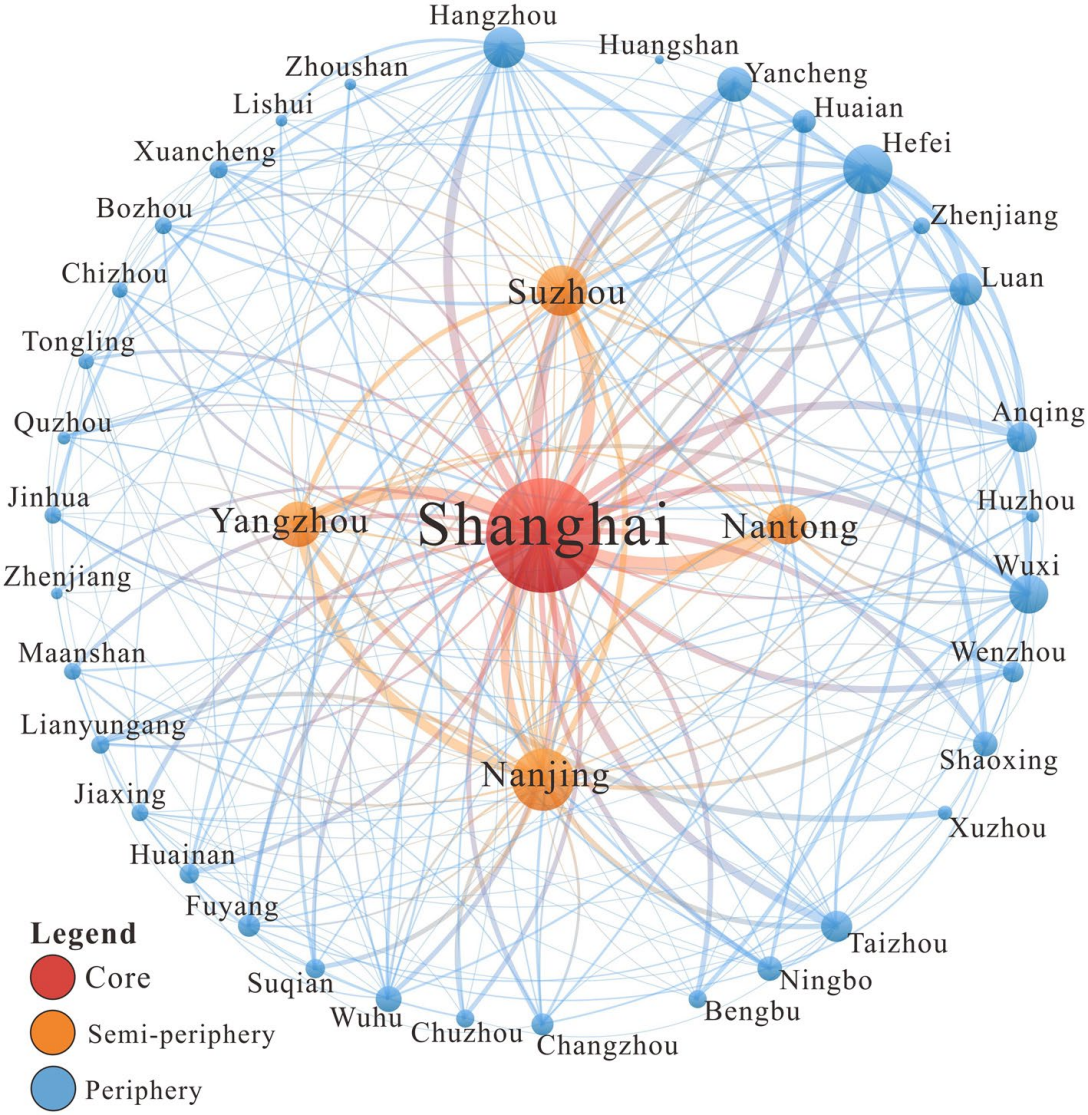
Core-periphery structure of the inter-city flow network of highly educated talents locally in the Yangtze River Delta.

The local flow network of highly educated talents in the Yangtze River Delta also presents a “pyramid-like” hierarchical structure, with an evident “core-periphery” hierarchical progressive form, which can be divided into three major urban groups: core area, semi-periphery area, and periphery area (Fig. 8).

Shanghai is the only core city in the inter-city flow network of highly educated talents in the Yangtze River Delta. It connects many cities and attracts a huge number of highly educated talents, so it is located at the center of the map. From a local perspective, Shanghai's network statistical metrics are far higher than the averages of the entire network (Table 3). The degree centrality of Shanghai is 43, and all cities in the network are connected with it. Furthermore, Shanghai has a weighted degree centrality of 587, accounting for 22.95% of the total value, and its weighted in-degree centrality is 573, accounting for 44.80% of the total. These rates indicate that on a local scale, Shanghai, as the top city in the Yangtze River Delta region, has attracted nearly half of the highly educated talents in the network.

**Table 3:** Characteristics of the hierarchical structure of the inter-city flow network of highly educated talents locally in the Yangtze River Delta.

Level	No. nodes	Average degree	Average weighted degree	Average closeness centrality	Average betweenness centrality	Density
Core	1	43.00	587.00	0.4375	280.73	0.4946
Semi-periphery	4	22.75	148.00	0.4826	95.97	0.2210
Periphery	36	12.24	40.56	0.4289	28.72	0.0857
Whole network	41	14.10	65.59	0.4346	42.08	0.1101

There are four cities in the semi-periphery area, namely Nanjing, Suzhou, Nantong, and Yangzhou. The network statistical metrics of this area are also higher than the averages of the entire network, with an average degree centrality of 22.75, indicating that the cities in this area are also closely connected with other cities in the region. Highly educated talents in this area flow frequently, with an average weighted degree centrality of 148.00. The net in-flow volume of highly educated talents in Nanjing is positive, and that of other cities is negative, indicating that although the cities in the semi-periphery area have all actively participated in the flow of highly educated talents, their roles are different. Nanjing is an in-flow city, while other cities are out-flow ones.

Most cities—a total of 36—are located at the periphery of the inter-city flow network of highly educated talents locally in the Yangtze River Delta, accounting for 87.80% of the total. These cities play an auxiliary role in the network and are less active. The Graph Density of the semi-periphery area is 0.0857, with an average degree centrality of 12.24, an average weighted degree centrality of 12.24, and an average betweenness degree centrality of 28.72. All the network statistical metrics are lower than the average. The periphery cities are mainly functioning to send highly educated talents to the core cities. There are 28 out-flow cities in this area, accounting for 77.78% of the total. Only Hangzhou, Hefei, Wuxi, and Xuzhou are somehow attractive, with a positive net in-flow volume of highly educated talents.

### The spatial characteristics of urban centrality from a local perspective

**Figure 9:**
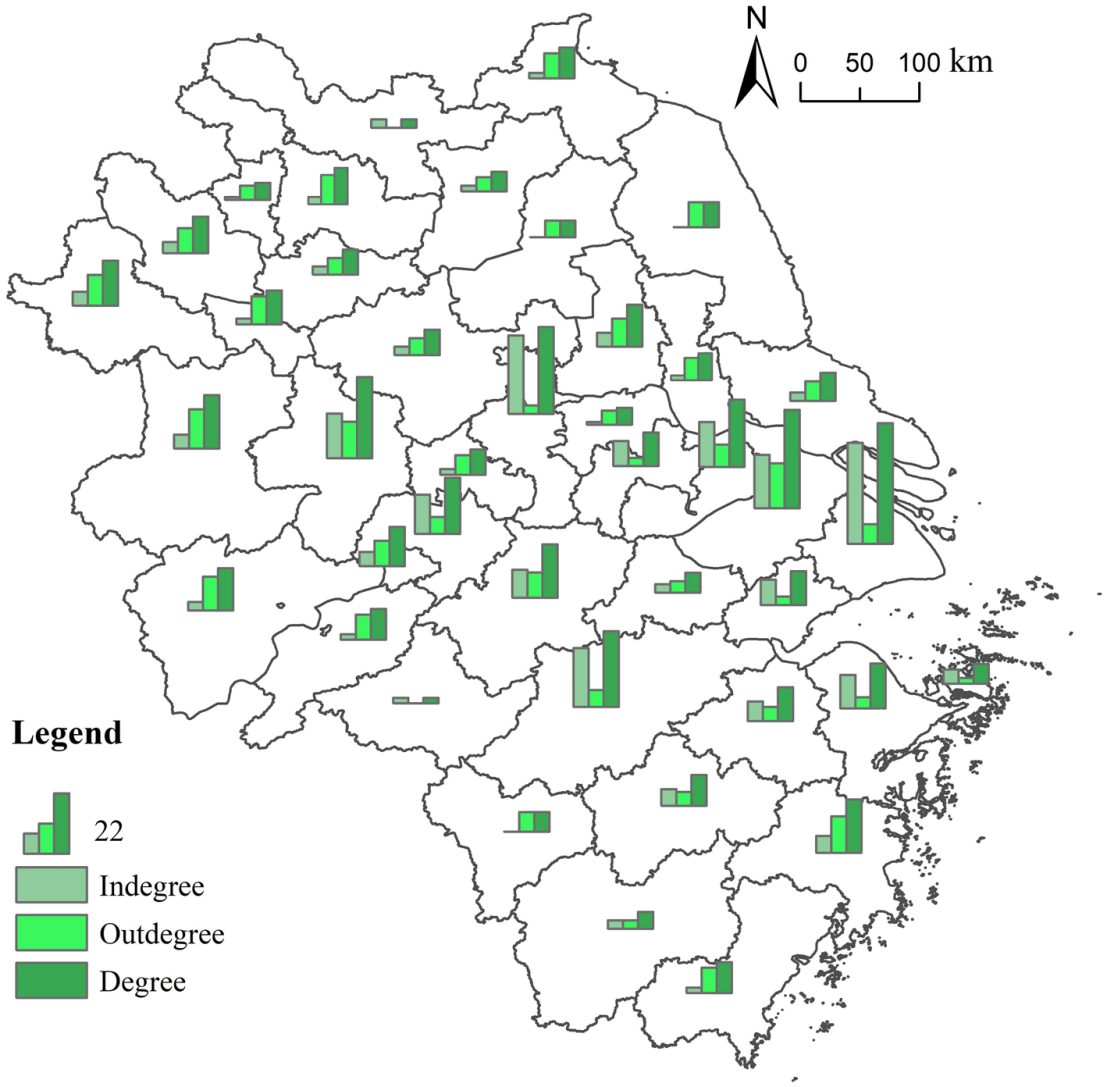
Degree centrality of nodes of the flow network of highly educated talents locally in the Yangtze River Delta. *Source*: Based on the standard map service website of the Ministry of Natural Resources, the approval number is GS (2020)3189. The base map boundary is not modified.

The high values of the degree centrality of nodes of the flow network of highly educated talents locally in the Yangtze River Delta showcase a “T” shape spatially distributed in the central and southern sections of the region (Fig. 9). In this “T” shape, the horizontal line is Lu’an-Hefei-Nanjing-Wuxi-Suzhou-Shanghai, and the vertical line is Wuhu-Xuancheng-Hangzhou-Taizhou. It can be observed that cities with higher administrative levels (e.g., Shanghai, Hangzhou, Nanjing, and Hefei) connect many other cities in the network, and the in-degree centrality of these areas is greater than their out-degree centrality. This means that they play the role of in-flow cities for highly educated talents in the network.

Furthermore, from the perspective of in-degree centrality, the high values of the nodes of the flow network of highly educated talents in the Yangtze River Delta are distributed in the central and eastern sections of the region. Shanghai and Nanjing have a relatively high in-degree centrality of 36 and 28, respectively. Hangzhou, Suzhou, Hefei, Wuxi, Wuhu, and Ningbo also have high values of in-degree centrality, which means that these cities are the in-flow places of highly educated talents from many other cities in the region. Contrary to the in-degree centrality, the high values of the out-degree centrality of nodes of the flow network of highly educated talents in the Yangtze River Delta are distributed in the periphery of the region, including Suzhou, Lu’an, Hefei, Taizhou, Anqing, Fuyang, Yangzhou, Huainan, and others. This distribution range is complementary to the high-value distribution range of high values of in-degree centrality, indicating that the cities in the periphery of the region are mainly out-flow cities for high-educated talents.

**Figure 10:**
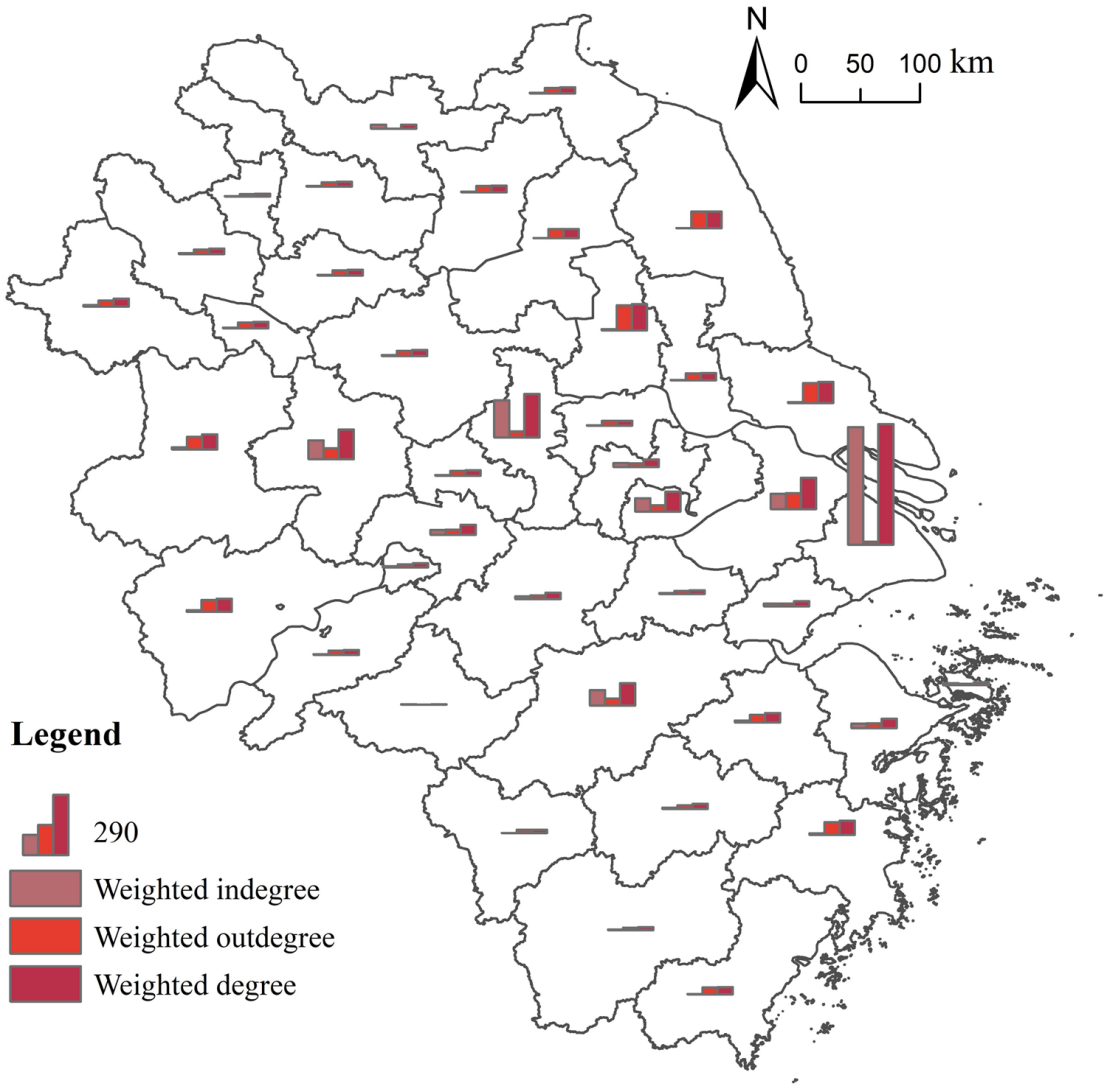
Weighted degree centrality of nodes of the flow network of highly educated talents locally in the Yangtze River Delta. *Source*: Based on the standard map service website of the Ministry of Natural Resources, the approval number is GS (2020)3189. The base map boundary is not modified.

There are significant differences in the weighted degree centrality of nodes of the flow network of highly educated talents locally in the Yangtze River Delta (Fig. 10). The high values of the weighted degree centrality present a zonal distribution from west to east along Hefei–Nanjing–Suzhou–Yangzhou–Hangzhou–Shanghai. Among them, Shanghai has the highest value (587), indicating that, as a regional leading city, it plays a pivotal role in the flow network of highly educated talents locally in the Yangtze River Delta.

From a local perspective, the weighted in-degree centrality of nodes of the flow network of highly educated talents follows an evident polarization. The weighted in-degree centrality of Shanghai is 573, accounting for 44.80% of the total, indicating that nearly half of the highly educated talents in the region flow to the city, making it the most important destination in that region. In addition, Nanjing, Hefei, Hangzhou, Suzhou, Wuxi and other economically developed cities also have high weighted in-degree centrality. The weighted in-degree centrality of the above seven core cities accounts for 84.68% of the total, showcasing that highly educated talents in the region have agglomerated in a small number of cities. Furthermore, the high-value areas of the weighted out-degree centrality are distributed in the border areas of the Yangtze River Delta, such as Yangzhou, Nantong, Yancheng, Suzhou, Lu’an, Taizhou, Anqing, Hefei, Huai'an, and Shaoxing, with an even spatial distribution. Similarly, the cities with high out-flow of highly educated talents in the Yangtze River Delta region are not the top cities in the region, but the ones distributed around the top region, which indicates that the top cities have strong factors that attract talents in their surrounding areas.

## Influencing factors of inter-city flow of highly educated talents in the Yangtze River Delta

Based on the “national-local” dual perspectives, the negative binomial regression model is used to explore the influencing factors on the inter-city flow network of highly educated talents between the Yangtze River Delta and the whole nation as well as those on the flow network of highly educated talents specifically concentrated in the Yangtze River Delta. This methodology was used to verify the six hypotheses proposed in Chapter 2 of this paper. In order to comprehensively analyze and compare the explanatory variables, the data used in this study was standardized by converting the original data into dimensionless index evaluation values. Prior to analysis, the model was tested for multicollinearity, and the criterion considered in this part of the process was VIF (Variance Inflation Factor): (i) maximum value of VIF is greater than 10 and (ii) average value of VIF is greater than 1^59^.

### The influencing factors of inter-city flow of highly educated talents in the Yangtze River Delta from a national perspective

The influence factors on the inter-city flow network of highly educated talents between the Yangtze River Delta and the whole nation were explored according to the aforementioned methodology. The multicollinearity test results showed that the average value of VIF is 2.43 and the maximum value is 5.81, indicating that there is no multicollinearity in the data. The results of the endogeneity test indicate that *p* is 0.8421, showing that the data does not have any endogeneity issues. The regression results are presented in Table 4.

**Table 4:** Regression results of factors influencing the inter-city flow of highly educated talents between the Yangtze River Delta and the whole nation.

Variables	Model 1	Model 2	Model 3	Model 4	Model 5	Model 6	Model 7
	*N*_*ij*_	*N*_*ij*_	*N*_*ij*_	*N*_*ij*_	*N*_*ij*_	*N*_*ij*_	*N*_*ij*_
*GDP*_*i*_	1.9727***						2.2325***
	(0.2058)						(0.1777)
*GDP*_*j*_	3.8307***						5.5882***
	(0.3104)						(1.5045)
*Colleges*_*j*_		1.4716***					− 1.5594
		(0.3425)					(1.0327)
*Universities*_*j*_		1.7313***					0.1183
		(0.2361)					(0.4554)
*Science*_*j*_			3.9801***				1.7424
			(0.3187)				(1.2336)
*Hospitals*_*j*_				3.0775***			− 0.5234
				(0.3812)			(1.1961)
*Buses*_*j*_				3.0748***			4.2741***
				(0.2798)			(0.9860)
*Greenbelt*_*j*_					− 0.0282		2.6559**
					(0.9431)		(1.1736)
*Pollution*_*i*_					0.5408		0.3322
					(0.3367)		(0.3027)
*Distance*_*ij*_						− 1.5619**	− 3.6134***
						(0.6494)	(1.0221)
*Province*_*ij*_						0.0000	0.0000
						(0.0000)	(0.0000)
Constant	− 5.9199***	− 4.9979***	− 5.5740***	− 5.0939***	− 3.1692***	− 2.5039***	− 6.6281***
	(0.2913)	(0.2254)	(0.2591)	(0.1793)	(0.6698)	(0.1886)	(0.6722)
lnalpha	− 32.7227	− 32.7227	− 32.7227	− 32.7227	− 32.7227	− 32.7227	− 32.7227
	(0.0000)	(0.0000)	(0.0000)	(0.0000)	(0.0000)	(0.0000)	(0.0000)
Observations	667	667	667	667	667	667	667
Wald chi2	188.30	145.52	155.99	225.07	2.58	5.79	375.98
Prob > chi2	0.0000	0.0000	0.0000	0.0000	0.2753	0.0162	0.0000
Log pseudolikelihood	− 101.75	− 110.94	− 107.49	− 111.40	− 128.41	− 127.89	− 97.79

Robust standard errors in parentheses, ***p < 0.01, **p < 0.05, *p < 0.1.

Hypothesis 1 is supported. The regional economic development situation affects the inter-city flow of highly educated talents between the Yangtze River Delta and the whole country. As shown in Model 1, the regression coefficient of the in-flow cities' GDP to highly educated talents is 3.8307, which means it passed the significance test of *p* < 0.01, indicating that the higher the economic level of the in-flow cities, the more in-flows of highly educated talents. The regression coefficient of the out-flow cities' GDP to highly educated talents is 1.9727, and it also passed the significance test of *p* < 0.01, proving that the higher the economic level of the out-flow cities, the more out-flows of highly educated talents. This result does not confirm hypothesis 1. The above regression results demonstrate that from the national perspective, the flow of highly educated talents tends to occur between economically developed cities. Cities with high economic levels show are highly attractive to talents since they can find more employment opportunities and higher salaries. Differently, in areas at high economic levels, talents have relatively more opportunities to go out to work for a long time, with more outing activities, such as exchanges, participation in conferences, visits, business meetings, etc., resulting in an active flow of these talents^54^. This result is different from the findings of previous research on the push-out effect of economic lag-behind regions on population^60^, indicating that compared to the flow of common population, that of talents has some special features.

Hypothesis 2 is supported. The educational development levels of cities affect the inter-city flow of highly educated talents between the Yangtze River Delta and the whole country, and the scale and quality of higher education in the in-flow cities has a positive impact on this flow. As seen in Model 2, the regression coefficient of the number of colleges and universities in the in-flow cities to the flow of highly educated talents is 1.4716, which means it passed the significance test of *p* < 0.01, indicating that the cities with more colleges and universities have a solid educational scenario, making them more attractive to highly educated talents. The regression coefficient of whether an in-flow city has first-class institutions of higher education to the flow of highly educated talents is 1.7313, so it also passes the significance test of *p* < 0.01 is passed. This indicates that the quality and level of higher education in the in-flow cities are important factors to be considered, and cities with first-class universities are hugely attractive to talents who want to continue their studies.

Hypothesis 3 is supported. The level of scientific and technological development impacts the flow of highly educated talents between the Yangtze River Delta region and other parts of the country, and the agglomeration of high-tech enterprises positively influences the volume of highly educated talents migration, meaning that the more high-tech enterprises a city attracts, the more highly educated talents it draws in. From Model 3, the regression coefficient of the number of high-tech enterprises in the in-flow city on the flow of highly educated talent is 3.9801, and this coefficient passes the significance test at *p* < 0.01, indicating that cities with a high level of high-tech development, characterized by a large number of high-tech enterprises, have a significantly stronger attraction for highly educated talents.

Hypothesis 4 is supported. Urban infrastructure conditions affect the inter-city flow of highly educated talents between the Yangtze River Delta and the whole country, and the completeness of urban infrastructure has a positive impact on the flow of highly educated talents. In other words, the more complete the infrastructure conditions of an in-flow city, the more highly educated talents it can attract. According to Model 4, the regression coefficient of the number of hospitals in the in-flow cities to the flow of highly educated talents is 3.0775. Since it passed significance test of *p* < 0.01, it is concluded that cities with superior medical and health conditions are significantly attractive to highly educated talents. Furthermore, the regression coefficient of the number of buses in the in-flow cities to the flow of highly educated talents is 3.0748, also passing *p* < 0.01, indicating that cities with functional transportation facilities are also targeted destinations for these talents.

Hypothesis 5 is not supported on the national scale. The urban environment does not affect the inter-city flow of highly educated talents between the Yangtze River Delta and the whole nation. The data showed that the aesthetics of the environment in an in-flow city and the environmental pollution in an out-flow city do not affect the flow of highly educated talents for the time being. Model 5 shows that the regression coefficient of the green coverage rate of in-flow cities’ built-up areas to the flow of highly educated talents is − 0.0282, but it fails to pass the significance test. The regression coefficient of PM 2.5 emissions in out-flow cities is 0.5408, which also fails the significance test. The possible reason behind this data is that there are small differences in green coverage rates of built-up areas of different cities, and environmental pollution is a common issue, so it can be challenging for talents to find a way to avoid it. However, Model 7 shows that the regression coefficient of the green coverage rate of urban built-up areas to the flow of highly educated talents is 2.6559. In this case, it passes the significance test of *p* < 0.01, indicating that the environmental conditions of cities also can positively affect highly educated talents when considering urban economy development, education, and infrastructure.

Hypothesis 6 is supported. Geographic distance negatively impacts on the inter-city flow of highly educated talents between the Yangtze River Delta and the whole country. In other words, the longer the distance of talents’ migration, the weaker the flow of talents between the cities. Model 6 shows that geographic distance is negatively correlated with the flow of highly educated talents. The regression coefficient of geographic distance is negative, so it passes the significance test of *p* < 0.01. In addition, in Model 7, geographic distance still has a negative impact on the inter-city flow of highly educated talents between the Yangtze River Delta and the whole country, indicating that the closer the geographic distance between the two cities, the greater the flow of highly educated talents. The data confirms that, at the national scale, geographical distance increases the cost in time, transportation, and economic burden to a certain extent, thus hindering the flow of highly educated talents. In addition, at the national scale, all cities are located in different provinces, so some cultural, institutional, and social form differences in talent migration are not verified.

### The influencing factors of inter-city flow of highly educated talents in the Yangtze River Delta from a local perspective

The influencing factors on the flow network of highly educated talents in the Yangtze River Delta were verified according to the aforementioned methodology. The multicollinearity test results showed that the mean value of VIF is 3.18 and the maximum value is 7.80, indicating that there is no multicollinearity in the data. In addition, the test result for endogeneity shows that p is 0.7892, showing that the data does not have any endogeneity issues. The regression results are shown in Table 5.

**Table 5:** Regression results of the influencing factors of the flow network of highly educated talents locally in the Yangtze River Delta.

*Variables*	Model 1	Model 2	Model 3	Model 4	Model 5	Model 6	Model 7
	*N*_*ij*_	*N*_*ij*_	*Nij*	*N*_*ij*_	*N*_*ij*_	*N*_*ij*_	*N*_*ij*_
*GDP*_*i*_	0.6646**						1.1618***
	(0.3175)						(0.3835)
*GDP*_*j*_	2.6113***						2.7176
	(0.2135)						(2.9961)
*Colleges*_*j*_		2.8013***					1.8344
		(0.3697)					(2.3127)
*Universities*_*j*_		0.3274					0.1803
		(0.2469)					(0.4954)
*Sciencej*			2.8749***				1.4895
			(0.2556)				(1.4768)
*Hospitals*_*j*_				0.1553			0.0759
				(0.8286)			(2.1510)
*Buses*_*j*_				2.7303***			− 0.8492
				(0.7870)			(4.5431)
*Greenbelt*_*j*_					2.9576***		0.8449
					(0.7066)		(0.9699)
*Pollution*_*i*_					− 0.0196		0.7319**
					(0.2480)		(0.3356)
*Distance*_*ij*_						− 0.9635	− 1.2836**
						(0.7727)	(0.6125)
*Province*_*ij*_						0.8820***	1.6854***
						(0.3294)	(0.2737)
Constant	− 4.1661***	− 4.7113***	− 4.4405***	− 4.3992***	− 1.8663***	− 2.2599***	− 7.1211***
	(0.1554)	(0.1957)	(0.1797)	(0.1658)	(0.3256)	(0.3961)	(0.8013)
lnalpha	− 31.5358	− 31.5358	− 31.5358	− 31.5358	− 31.5358	− 31.5358	− 31.5358
	(0.0000)	(0.0000)	(0.0000)	(0.0000)	(0.0000)	(0.0000)	(0.0000)
Observations	283	283	283	283	283	283	283
Wald chi2	149.56	140.10	126.48	153.00	17.72	8.85	352.04
Prob > chi2	0.0000	0.0000	0.0000	0.0000	0.0001	0.0120	0.0000
Log pseudolikelihood	− 44.59	− 43.71	− 44.67	− 44.20	− 50.46	− 51.83	− 41.20

Robust standard errors in parentheses, ***p < 0.01, **p < 0.05, *p < 0.1.

Hypothesis 1 is supported. The regional economic development situation affects the inter-city flow network of highly educated talents locally in the Yangtze River Delta. Model 1 shows that the regression coefficient of in-flow cities’ GDP to highly educated talents is 2.6113, so it passes the significance test of *p* < 0.01. This indicates that the higher the economic level of an in-flow city, the more in-flows of highly educated talents. The regression coefficient of out-flow cities' GDP to highly educated talents is 0.6646, also passing the significance test of *p* < 0.05 and indicating that the higher the economic level of an out-flow city, the more out-flows of highly educated talents. This result is consistent with the situation at the national scale.

Hypothesis 2 is supported. The educational development level of cities affects the inter-city flow of highly educated talents locally in the Yangtze River Delta, and the scale of higher education in the in-flow cities has a positive impact on the flow of highly educated talents. Model 2 shows that the regression coefficient of the number of colleges and universities in the in-flow cities to the flow of highly educated talents is 2.8013. The data pass the significance test of *p* < 0.01, indicating that on a local scale, the level and diversity of higher education are significantly attractive to highly educated talents. The regression coefficient of whether the in-flow cities have first-class institutions of higher education to the flow of highly educated talents is 0.3274, so it fails the significance test. This indicates that within the scope of the Yangtze River Delta, the level of the in-flow cities’ higher education does not play a role in the flow of highly educated talents. The reason for this result may be that there is little difference in the level of higher education among cities in the region.

Hypothesis 3 is supported. The scientific and technological development level impacts the intraregional intercity flow of highly educated talents within the Yangtze River Delta region, and the concentration of high-tech enterprises positively affects the volume of highly educated talents migration. Specifically, the more high-tech enterprises a destination city hosts, the greater the number of highly educated talents it attracts. From Model 3, the regression coefficient of the number of high-tech enterprises in the in-flow city on the flow of highly educated talent is 2.8749, and this coefficient passes the significance test at *p* < 0.01, indicating that cities with high levels of high-tech development at the local scale still have a significant attraction to highly educated talents.

Hypothesis 4 is supported. The urban infrastructure conditions affect the inter-city flow of highly educated talents locally in the Yangtze River Delta. The completeness of cities’ infrastructure has a positive impact on the flow of highly educated talents. The better the infrastructure of an in-flow city, the more highly educated talents it will attract. As shown in Model 4, the regression coefficient of the number of hospitals in the in-flow cities to the flow of highly educated talents is 0.1553, which means it fails the significance test. However, the regression coefficient of the number of buses in the in-flow cities to the flow of highly educated talents is 2.7303, passing the significance test of *p* < 0.01. This outcome confirms that cities with functional transportation facilities are significantly attractive to highly educated talents.

Hypothesis 5 is supported. The urban environment affects the flow of highly educated talents locally in the Yangtze River Delta. The aesthetics of the environment in the in-flow cities have a positive effect on the flow of talents in the Yangtze River Delta. As shown in Model 5, the regression coefficient of the green coverage rate of urban built-up areas to the flow of highly educated talents is 2.9576, so it passes the significance test of *p* < 0.01, demonstrating that the visual quality of the environment can strengthen the in-flow of highly educated talents. This means that areas with larger urban green spaces are more likely to attract talents. The regression coefficient of PM 2.5 emission of the out-flow cities does not pass the significance test, indicating that the environmental pollution of out-flow cities currently does not play a role in the flow of highly educated talents in the Yangtze River Delta. However, as shown in Model 7, if other factors such as the city’s economy and education level are considered, the regression coefficient of PM 2.5 emission of out-flow cities is positive, and it passes the significance test. This indicated that areas with severe environmental pollution cause a “push-out” effect on these talents.

Hypothesis 6 is supported. Geographical distance has a negative impact on the inter-city flow of highly educated talents between the Yangtze River Delta and the whole country. Model 6 shows that geographic distance is negatively correlated with the flow of highly educated talents, failing the significance test. However, Model 7 shows that if other factors are considered, geographic distance would cause a negative effect on the flow of highly educated talents. In other words, the greater the geographic distance between two cities, the weaker the flow of highly educated talents between them.

Hypothesis 7 is supported. The smaller the gaps in culture, institution, and social form between the in-flow and out-flow places, the more frequent the flow of talents between them. As shown in Model 6 and 7, if the out-flow and in-flow areas are located in the same province, the flow of highly educated talents is strengthened. Their regression coefficient is 0.8820 and 1.6854, respectively, so both pass the significance test of *p* < 0.01. This outcome indicates that similar institutions, cultures, customs, and languages can reduce the resistance of highly educated talents to reach their destination cities. It was also concluded that highly educated talents in the Yangtze River Delta are more inclined to flow within the same province.

## Conclusion and discussion

Based on China's population dynamic monitoring data and using a combination of complex networks, spatial analysis, mathematical measurement, and other related theories and methodologies, this study described the flow network of high education talents in the Yangtze River Delta from national and local perspectives. Considering the dimensions of overall flow characteristics, core-periphery structure, node centrality, this study also revealed the characteristics of the flow network of highly educated talents in the Yangtze River Delta in the two perspectives. At the same time, this paper explored the influencing factors of the flow of highly educated talents in the Yangtze River Delta in the two perspectives. The main conclusions are summarized below.

In both perspectives, the flow network of high education talents in the Yangtze River Delta exhibits significant spatial heterogeneity and hierarchical structure. The main trend in this network is characterized by the flow of such talents towards national core cities. From a national perspective, the network has formed a spatial pattern in which the highly educated talents across the country converge to the Yangtze River Delta. The Yangtze River Delta is an important in-flow region of highly educated talents in the country. Shanghai as the top city in the region is the most important destination for the flow of highly educated talents. While most cities outside the region do not have a lot of factors that can attract this kind of talents in the Yangtze River Delta, who mainly prefer to reallocate to first-tier cities in the country. From a local perspective, the inter-city flow network of highly educated talents in the Yangtze River Delta has a close connection, which is characterized by high density and high connectivity. This means that highly educated talents in the region converge to a few cities, and the economically developed cities have a higher weighted in-degree centrality. The in-flow of highly educated talents from the provincial capital cities with higher administrative levels and the economically developed cities to Shanghai constitutes the backbone of the network, with a convergent spatial trend. Both the inter-city flow network of highly educated talents between the Yangtze River Delta and the whole nation and the inter-city flow network of highly educated talents locally in the Yangtze River Delta have developed a “core-periphery” hierarchical progressive form, with Shanghai as the core city. As the leading city in the Yangtze River Delta region, Shanghai maintains connections with numerous cities and exerts a pronounced “gravitational pull” or “talent suction effect” on highly educated talents across the nation and within the region itself.

Multiple factors comprehensively affect the flow pattern of highly educated talents in the Yangtze River Delta. The economic development status of cities has a deep-rooted impact on the flow of highly educated talents. Many noneconomic factors also influence this flow, including urban education levels, scientific and technological levels, infrastructure condition, regional environment, geographic distance, cultural identity, institutional gaps, and social form differences. At a broader national scale, whether a city can successfully attract and retain highly educated talent depends not only on its macroeconomic strength but also critically on the provision of high-quality educational resources and support from scientific and technological research enterprises. Moreover, it is closely related to the level of sophistication of the city's infrastructure construction. Cities with high levels of economic development typically possess stronger agglomeration effects, offer higher salary packages, and present more extensive career advancement opportunities—all of which are critical factors in attracting highly educated talents. For those who pursue further academic research or aspire to develop deeply in specific domains, the quality and quantity of higher education institutions and technology companies within a city directly influence the potential for career progression. Infrastructure conditions rank as one of their significant considerations. Cities equipped with superior transportation and medical infrastructure are more likely to attract the inflow of highly educated talents. At the mesoscale regional level, cities within the Yangtze River Delta region may exhibit certain degrees of synergy and convergence in terms of economic development, education standards, and technological reserves. However, this does not imply that all cities within the region have an equal attraction for the mobility of highly educated talents. Even within the same region, different cities can exert varying impacts on the flow of such talents. On the one hand, highly educated talents indeed pay attention to the overall level of regional economic development, which determines the scope of potential job opportunities and career advancement they might encounter. On the other hand, the status of education and technology development is also a critical consideration, with the strength of higher education and high-tech capabilities serving as a platform for talent to showcase their abilities and potentially influencing the educational environment for their children. Beyond these fundamental factors, highly educated talents place greater emphasis on the quality of life, which encompasses multiple aspects such as urban environmental quality, cost of living, public service facilities (e.g., healthcare, culture, and entertainment), humanistic atmosphere, and pace of life. A high quality of life not only ensures that they can obtain adequate rest and enjoyment outside of work but is also instrumental in realizing their self-worth and enhancing their sense of happiness, being key to their overall well-being and contentment.

It is worth noting that while economically developed areas boast numerous advantages that attract talent inflow, concurrently, due to their highly active economic environment, these regions often come with intense occupational competition pressure, limited career advancement opportunities, and rapidly escalating living costs, among other issues. Against this backdrop, talent migration takes into account a variety of factors, such as future family living standards, government guidance and preferential policies, as well as sentiments attached to returning to one's hometown. Consequently, in regions with higher economic levels, there is also a relatively frequent phenomenon of talent outflow, which distinctly contrasts with the characteristics of ordinary labor force mobility. Moreover, at both scales, geographical distance remains a significant factor affecting the flow of highly educated talent in the Yangtze River Delta region. Generally, the farther the distance of talent migration, the less the volume of talent flow between regions. When considering relocation, talent tends to weigh up the costs involved, which include psychological costs and actual expenses stemming from physical distance, such as commuting time and migration expenses. The farther the distance, the greater the resistance to talent migration could become. Proximity in terms of systems, culture, customs, and language can mitigate the barriers faced by talent upon reaching their destination. In practical talent movements, highly educated talents tend to favor relocating within the same province or to adjacent cities, as this type of migration not only reduces migration costs but also enables them to leverage existing social networks to quickly find platforms suitable for their personal and professional development. This proximity effectively minimizes the adaptation costs and psychological barriers during the talent flow process, allowing individuals to integrate more swiftly into the local society and workplace environment upon arriving at their new location, thereby increasing job satisfaction and overall life happiness.

There is frequent talent interaction between the national and Yangtze River Delta region, and the country can effectively promote mutual engagement and circulation of talent between the two, implementing a series of coordinated measures to facilitate efficient talent mobility and rational allocation. At the national level, unified standards and policies should be established for talent evaluation, selection, cultivation, utilization, and mobility, breaking down regional barriers and ensuring that talents enjoy fair competition and development opportunities nationwide. Efforts should be made to establish and improve mechanisms for talent mobility, simplifying procedures for cross-regional talent transfers, refining household registration, social insurance, and housing provident fund transfer policies, guaranteeing that the rights and interests of talents are not compromised during their movement. The country encourages collaboration between the Yangtze River Delta and other regions on talent exchange programs, promoting interaction and diffusion of knowledge and technology through means such as rotational postings, temporary training assignments, and joint research and development projects. Special funding schemes should be set up to support cross-regional talent team collaborations, fostering technological innovation and industrial upgrading. Aligned with the nation's major strategic development plans, the country guides talents towards areas and fields with urgent and scarce needs. This includes encouraging high-end talents in the Yangtze River Delta region to share experiences and technologies with Central and Western regions, while simultaneously encouraging talents from those regions to receive training and further education in the Yangtze River Delta.

The Yangtze River Delta is the earliest region in China to initiate regional integration and has accumulated the strongest foundation for cross-regional coordination mechanisms. Since the integration of the Yangtze River Delta has been elevated to a national strategy, talent integration serves as a critical supporting mechanism for achieving regional integration within the area. The Yangtze River Delta region should strive to become a model for talent integration both domestically and globally. The region should establish a cross-regional talent policy coordination mechanism to reduce institutional barriers to talent mobility. It should design and implement talent incentive policies to attract and retain highly educated talents. Optimizing the allocation of public service resources within the region, such as education, science and technology, healthcare, and elderly care, aims to realize the sharing and circulation of public services, addressing concerns following talent mobility. Strengthening the construction of transportation networks will enhance the accessibility and convenience of internal regional transportation, shortening spatial and temporal distances between cities and facilitating talent flow. Building a unified talent information platform and service system allows for the sharing of educational resources and research facilities, encouraging cross-regional cooperation in industry-university-research projects. Creating a first-class environment for talent development, encompassing high-quality living and working conditions, is essential. By leveraging its economic, technological, educational, and cultural advantages, the Yangtze River Delta region can better form a talent concentration hub, driving efficient and optimized flow and distribution of talent resources within the area.

This study has several limitations that can be addressed in future research. Firstly, the data concerning highly educated talents utilized in this investigation was derived from dynamic monitoring of migrant populations, which is a conventional method when examining the distribution or mobility of such talents. However, the representativeness of this data may be affected by various factors, such as potential sampling errors inherent in the data collection process. The reliability of migrant population data heavily relies on the effectiveness of registration systems, and given the typically higher mobility characteristic of highly educated talents, a one-time survey or periodic monitoring may fail to capture the full spectrum of shifts occurring. Secondly, while macro-level city factors are indeed crucial in analyzing the influential factors of highly educated talent mobility, individual variations and intrinsic motivations should not be disregarded. Factors like personal career planning, family background, life aspirations, cultural adaptability, and interpersonal networks can profoundly shape the migration decisions of these talents. Additionally, certain macroscopic elements, like policy factors, which are identified as key influencers of talent mobility, are difficult to evaluate quantitatively. While policy influences have been discussed in this study, talent policies encompass a wide array of aspects including talent recruitment, career progression, social security, regional development, and cultural environments. The actual impact of these policies can extend to subjective realms like individual perception, psychological reactions, and anticipated gains, as well as objective aspects like policy execution efficiency, fairness, and sustainability—all of which are challenging to assess precisely through a single quantitative metric. Thus, to gain a comprehensive understanding of the complex phenomenon of talent mobility, future research can employ a mixed-methodological approach that both focuses on macro-trends via big data analysis and delves into microscopic perspectives through in-depth methods like interviews, questionnaire surveys, and case tracking. Moreover, talent mobility is a dynamic process that evolves over time and in response to changing socio-economic conditions, potentially exhibiting significant heterogeneity in its spatial evolution and driving mechanisms. Due to data constraints, this study did not explore evolutionary dynamics. As such, future endeavors should undertake time-segmented and phased research, contrasting historical data longitudinally to scrutinize the distinctive characteristics of highly educated talents mobility across different periods and the shifts in the underlying driving forces.

## Data Availability

All data generated or analysed during this study are included in this published article and its supplementary information files.
